# Necrotizing Enterocolitis: The Role of Hypoxia, Gut Microbiome, and Microbial Metabolites

**DOI:** 10.3390/ijms24032471

**Published:** 2023-01-27

**Authors:** Aleksandra Kaplina, Svetlana Kononova, Ekaterina Zaikova, Tatiana Pervunina, Natalia Petrova, Stanislav Sitkin

**Affiliations:** 1 Research Laboratory for Physiology and Diseases of Newborns, Almazov National Medical Research Centre, St. Petersburg 197341, Russia; 2 Group of Protein Synthesis Regulation, Institute of Protein Research, Russian Academy of Sciences, Pushchino 142290, Russia; 3 Research Laboratory of Autoimmune and Autoinflammatory Diseases, Almazov National Medical Research Centre, St. Petersburg 197341, Russia; 4 Institute of Perinatology and Pediatrics, Almazov National Medical Research Centre, St. Petersburg 197341, Russia; 5 Epigenetics and Metagenomics Group, Institute of Perinatology and Pediatrics, Almazov National Medical Research Centre, St. Petersburg 197341, Russia; 6 Department of Internal Diseases, Gastroenterology and Dietetics, North-Western State Medical University Named after I.I. Mechnikov, St. Petersburg 191015, Russia

**Keywords:** necrotizing enterocolitis, NEC, hypoxia, glyco-redox, gut microbiome, microbial metabolites, fucose

## Abstract

Necrotizing enterocolitis (NEC) is a life-threatening disease that predominantly affects very low birth weight preterm infants. Development of NEC in preterm infants is accompanied by high mortality. Surgical treatment of NEC can be complicated by short bowel syndrome, intestinal failure, parenteral nutrition-associated liver disease, and neurodevelopmental delay. Issues surrounding pathogenesis, prevention, and treatment of NEC remain unclear. This review summarizes data on prenatal risk factors for NEC, the role of pre-eclampsia, and intrauterine growth retardation in the pathogenesis of NEC. The role of hypoxia in NEC is discussed. Recent data on the role of the intestinal microbiome in the development of NEC, and features of the metabolome that can serve as potential biomarkers, are presented. The *Pseudomonadota* phylum is known to be associated with NEC in preterm neonates, and the role of other bacteria and their metabolites in NEC pathogenesis is also discussed. The most promising approaches for preventing and treating NEC are summarized.

## 1. Introduction

Necrotizing enterocolitis (NEC) is a severe disease that predominantly affects preterm infants with a birth weight of less than 1500 g, and the incidence ranges from 2% to 13% [1,2]. NEC in preterm infants is associated with a high mortality rate (20–30%) [1], which increases to 50% in extremely low body weight (ELBW) neonates undergoing surgical treatment for NEC [3]. In some cases, NEC can affect full-term infants with congenital heart disease (CHD) [4], as well as neonates with perinatal asphyxia, polycythemia/thrombotic conditions, endocrine diseases, and perinatal sepsis [5,6].

NEC has multifactorial causes [7] and is associated with various prenatal and postnatal factors. The complexity of detecting clinical signs and a lack of reliable early diagnostic markers make NEC difficult to diagnose in a timely manner and differentiate from other neonatal complications [8]. However, the roles of some factors in the pathogenesis of NEC are known, including genetic predisposition [6,9], prematurity, enteral formula feeding, vasoactive and inflammatory mediators and the role of enteral antibiotics [7]. Over the past decade, knowledge on immune and digestive system development in children has advanced [10,11]. Our understanding of gut microbiota formation in children [12] and how the mother’s microbiome and external factors influence it has broadened. Similarly, we now understand in more detail how changes in cellular redox state are related to the glycosylation of proteins and lipids in various biological processes [13]. The presence of certain glycan patterns in the mucins and glycocalyx of endometrial cells is necessary not only for proper blastocyst implantation and subsequent developmental phases, but also for vascularization, placental development and creating an immunomodulatory environment for maternal tolerance to fetal antigens [14]. In addition, the glycan profile of mucins, the glycocalyx of intestinal epithelial cells, and breast milk oligosaccharides also influence the shaping of the intestinal microbiome [15]. These data also allow looking at the molecular mechanisms of the pathogenesis of NEC from the positions of glycobiology, similar to what happened in our understanding of inflammatory bowel disease (IBD) [16].

Research on the pathophysiology of NEC suggests that the lipopolysaccharide (LPS) of gut-colonizing bacteria binds to Toll-like receptor 4 (TLR4) on intestinal epithelial cells and triggers a process leading to enterocyte apoptosis and disruption of the intestinal epithelial barrier [17]. This allows bacteria to access the underlying tissues and leads to an intense inflammatory response mediated by tumor necrosis factor-alpha (TNF-α), interleukin-1β (IL-1β) and other inflammatory cytokines [6,18]. In addition, bacteria translocating from the intestinal lumen interact with TLR4 on the lining of mesenteric blood vessels, which is accompanied by vasoconstriction and intestinal ischemia [17]. Impairment of blood flow autoregulation in preterm infants in response to hypoxia, an imbalance between the production of endothelin-1 (ET-1) and nitric oxide (NO) in the intestine of newborns, exacerbates intestinal ischemia [19]. Platelet-activating factor (PAF), produced by platelets, inflammatory cells (neutrophils, eosinophils, and macrophages), and some bacteria (*Escherichia coli* and *Helicobacter pylori*), also plays a role in intestinal injury induced by hypoxia/reperfusion, TNF-α and LPS, leading to intestinal necrosis [18].

In this review, we discuss the role of hypoxia and the mother and infant intestinal microbiomes in the development of NEC, and how this may be related to milk oligosaccharides metabolized by the infant microbiota.

## 2. Risk Factors for NEC

### 2.1. Prenatal Risk Factors

Risk factors of NEC can be divided into those associated with hypoxia (limitation of blood oxygen delivery and malnutrition of the fetus) and those related to microbiome features. The most significant prenatal NEC risk factors associated with intrauterine hypoxia and impaired tissue perfusion include abnormal blood flow in the umbilical artery [20], especially lack of blood flow/ reversible end-diastolic blood flow [21], fetal distress [22], maternal hypertension [23] and placental abruption [24]. The cause of the absence of/end-diastolic blood flow in the umbilical artery (absent or reversed end-diastolic velocity) is chronic placental insufficiency [25], which can be induced by pre-eclampsia (PE) and maternal hypertension [26].

In addition to PE, gestational diabetes mellitus (GDM), maternal malnutrition, excessive alcohol consumption, smoking and glucocorticoid treatment during pregnancy can also lead to intrauterine growth retardation (IUGR). IUGR [20] and lower birth weight [22,23,27] can also be the result of intrauterine hypoxia (impaired placental blood flow). The main cause of IUGR is placental insufficiency associated with increased oxidative stress in the placenta [28]. According to Ree et al. (2014), preterm infants with IUGR are twice as likely to develop NEC compared to preterm infants appropriate for gestational age (GA) [29].

Yang et al. (2018) showed the role of pregnancy-induced hypertension as a risk factor for NEC [30]. Uteroplacental insufficiency secondary to pregnancy-induced hypertension can lead to fetal hypoxia and the release of proinflammatory cytokines [30].

Data on the role of PE as a risk factor of NEC are controversial. Ahle et al. (2018) found a negative correlation between maternal PE and the development of NEC in neonates [22]. However, Duci et al. (2019) reported that maternal PE in combination with lower birth weight is a risk factor of NEC [20]. Neonates of mothers with PE have a 2.5-fold increased risk of NEC [24]. This heterogeneity of data on how maternal PE and birth weight influence the development of NEC may be due to differences in the GA of preterm infants.

The main cause of induced preterm labor is impaired placental blood flow. However, one of the reasons for spontaneous preterm labor [31], and an NEC risk factor [20], is infectious and inflammatory lesions of the placenta and intrauterine infection of the fetus [31]. Several studies on pregnant animals have shown that maternal inflammation affects the fetus and causes intestinal damage, elevated serum levels of inflammatory cytokines, and loss of Paneth-cell and goblet-cell density; it is believed that the inflammatory cascade itself may be of decisive importance in the development of neonatal complications [32].

Additionally, there is controversy regarding the risk factors of NEC associated with infection [20]. A number of studies unambiguously attributed chorioamnionitis in the mother (especially when confirmed histologically) [20] and the premature rupture of membranes as risk factors [24,27]. However, Ahle et al. (2018) found negative associations between maternal urinary tract infection, premature rupture of membranes, and the development of NEC [22]. Meanwhile, Tan et al. (2022) found no association between the premature rupture of membranes and the development of NEC [33].

### 2.2. Perinatal and Postnatal Risk Factors of NEC

There is a known association between lower GA at birth and the development of NEC [23,27,34], but the risk factors of NEC according to GA are variable [22]. In neonates with a GA of 28-31 weeks, NEC was associated with isoimmunization, fetal distress, persistent ductus arteriosus (PDA), bacterial infection/sepsis, and red-blood-cell transfusion [22]. CHD, birth by caesarean section, the presence of chromosomal abnormalities, and an Apgar score < 7 at 5 min were related to NEC in neonates with a GA > 31 weeks [22]. In a systematic review, Samuels et al. (2017) identified low birth weight, a low GA, sepsis, assisted ventilation, arterial hypotension, the premature rupture of membranes, and black ethnicity as risk factors of NEC [27].

Wang et al. (2022) reported other postnatal risk factors of NEC including grade ≥ 2 intracranial hemorrhage, peripherally placed central catheterization, breast milk enrichment, red-blood-cell suspension transfusion, hematocrit > 49.65%, mean corpuscular volume > 114.35 fl, and mean platelet volume > 10.95 fl [35]. A summary of risk factors for NEC is presented in Table 1.

## 3. The Role of Hypoxia as a Risk Factor for NEC

NEC in term infants is thought to have a hypoxic-ischemic etiology [4] but the pathophysiology of NEC in preterm infants is mainly associated with intestinal immaturity and an aberrant microbiome [10]. A comparison of the location of intestinal lesions and the time of NEC onset in term and preterm infants revealed that in term infants, NEC develops in the first week of life and is located mainly in the proximal colon, while in preterm infants, it is mainly in the distal ileum [4] and ileocecal area [6]. This variation in NEC localization is explained by differences in blood supply and the degree of ischemia in these parts of the intestine due to the location at watershed areas of perfusion by the superior and inferior mesenteric arteries [49]. Surmeli Onay et al. (2020) proposed differentiating NEC in preterm infants with prenatal hemodynamic events (maternal hypertension and abnormal blood flow in the umbilical artery) and IUGR in combination with an early onset of NEC (before 7 days of life) as hypoxic-ischemic enterocolitis [50]. However, consideration of NEC features showed that the role of hypoxia in the development of NEC is not limited to the postnatal period.

### 3.1. Physiological Niches of Hypoxia: Fetus, Placenta, and Intestines

Although most tissues in the body are provided with sufficient oxygen to meet the metabolic and bioenergetic needs of cells through the capillary network, there are tissues where so-called physiological hypoxia is a normal phenomenon, providing the metabolic and energy processes necessary for cell functions [51]. Such areas include, in particular, the placenta and fetus, and intestinal epithelial cells. The maintenance of physiological hypoxia is a tightly controlled process, but in pathological niches such as chronic tissue inflammation, oxygen gradients are often more chaotic and less stable, leading to inappropriate levels of reactive oxygen species (ROS) and provoking oxidative stress [51].

In the placenta and the fetus, the level of hypoxia changes during pregnancy. Normally, during the first trimester of pregnancy, hypoxia is a physiological condition that ensures successful placentation. Blood flow through the maternal spiral arteries in the intervillous space begins between the 10th and 12th weeks of gestation, and oxygen partial pressure gradually increases during normal pregnancy. This process correlates with successful pregnancy outcomes. In the case of miscarriage, maternal-placental blood flow was established approximately 2 weeks earlier. In the case of abnormal differentiation of the trophoblast in the first trimester due to decreased endometrium invasion and a decrease in remodeling of the maternal spiral arteries, uteroplacental perfusion insufficiency develops. This results in episodes of hypoxia or hypoxia/reoxygenation [28,52]. Lien et al. (2021) revealed mitochondrial dysfunction in placental cells in spontaneous preterm birth [53]. In mitochondria, oxidative phosphorylation was impaired, which led to changes in energy metabolism and cell homeostasis, responses to oxidative stress, and the triggering of inflammatory pathways. Triggered inflammatory processes can lead to the development of PE and IUGR in the third trimester of pregnancy or earlier [28,52]. PE may or may not be associated with fetal growth retardation, but the combination of these two factors increases the risk of NEC by more than 3-fold [32].

The rearrangement of metabolic processes in the cell during hypoxia is regulated by transcription factors induced by hypoxia (HIF)—a dimer assembled from α (HIF-1α, HIF-2α or HIF-3α) and β (HIF-β) subunits. HIF containing either HIF-1α or HIF-2α subunits activates more than 500 genes expressed in response to tissue hypoxia and ischemia, suppressing the transcription of many genes functioning under normoxia conditions. HIF-1α rearranges cell bioenergetics by promoting glycolysis and suppressing oxidative phosphorylation; HIF-1α and HIF-2α also promote angiogenesis, which is regulated by vascular endothelial growth factor (VEGF), and they trigger the expression of genes that provide the barrier function of the intestinal mucosa [51]. HIF-1α also regulates the expression of many genes required for placental vascular morphogenesis and trophoblast differentiation. During the development of the embryonic vascular system, VEGF signaling occurs in endothelial cells through the vascular endothelial growth factor receptor (VEGFR), which is necessary to direct the entry of microvessels into the lamina propria during the development of villi and the creation of the intestinal mucosal microvasculature [51]. In mice, during the last three days of pregnancy, VEGF and VEGFR2 are highly expressed in the fetal intestine, and after delivery their expression is markedly reduced [49]. This is believed to ensure the proper development of the fetal intestinal microvasculature as the due delivery date approaches. Inappropriate timing of the decrease in HIF-mediated signaling may abolish VEGF production during a critical period in the development of the intestinal microvasculature, predisposing very-preterm infants to NEC due to its underdevelopment [49].

In the digestive tract, as it is colonized by anaerobes, there is a longitudinal (from the small intestine to the large intestine) and radial (from the lamina propria to the intestinal lumen) spatial oxygen gradient [54]. In the small intestine, the base of the villi is better oxygenated, and the tips are hypoxic due to dynamic and rapid fluctuations in cellular oxygen tension [54]. Maintenance of the oxygen gradient is provided by the oxygen metabolism of epithelial and subepithelial cells during food digestion and nutrient absorption, as well as by the gut microbiota in the lumen. The energy needs of intestinal epithelial cells are provided by oxidative phosphorylation in mitochondria; therefore, the level of oxygen in cells is tightly regulated, and excessive fluctuations can provoke pathology [54]. In turn, gut oxygenation directly affects the composition of gut microbial communities and oxidative changes in gut inflammation. The microbial content and composition increase along the longitudinal axis of the intestine and between the apex and base of the intestinal epithelium villi. Short-chain fatty acids (SCFAs) produced by the colonic microbiota, especially butyrate, stimulate epithelial metabolism and increase epithelial oxygen consumption to a level that cells perceive as metabolic hypoxia, which leads to HIF stabilization [54].

### 3.2. Oxidative Stress and Its Consequences for Glycosylation of the Cellular Glycocalyx

Oxidative stress provoked by pathological hypoxia can lead to dysfunction of several glycosyltransferases localized in the Golgi apparatus and, as a result, to disruption of the glycan profile of the cell glycocalyx. Currently, there are a limited number of reports on the regulation of glycosyltransferase gene expression by oxidative stress. In particular, upregulation by oxidative stress of gene expression of fucosyltransferase VII (FUT7); sialyltransferase ST3Gal-I; uridine diphosphate glucuronosyl transferase (UGT1), and transporters of uridine diphosphate galactose (UDP-galactose), and sialic acid (sialin) has been reported [13]. By contrast, HIF-1α suppressed the gene expression of α1,2-fucosyltransferase (FUT1 and FUT2) in the pancreatic cancer human cell lines Pa-Tu-8988S and Pa-Tu-8988T [13]. In the latter case, different levels of HIF-1α expression in different cell lines not only affected the degree of α1,2-fucosylation by FUT1 and FUT2 fucosyltransferases, but also the invasive ability of cells [55].

During pregnancy, complex interactions occur between the tissues of the mother and the fetus, ensuring the successful implantation and development of the embryo, including through the development of a functional vascularized placenta and the establishment of immune tolerance. During these processes, dynamic changes occur in various glycan epitopes of the glycocalyx of syncytiotrophoblast cells in the maternal decidua [56]. Some pregnancy complications such as PE disrupt glycosylation, including sialylation and fucosylation [56,57,58].

The expression of glycosyltransferases responsible for the synthesis of epitopes carrying N-acetyl-glucosamine (GlcNAc), sialic acid, or fucose (Fuc) changes throughout pregnancy [14]. The expression of some of these enzymes is controlled, in particular, by changing the oxygen tension. These changes are an important part of regulatory processes. Redox imbalance in placental malperfusion leads to the impaired expression of glycan patterns of the glycocalyx of placental barrier cells [57], including syncytiotrophoblast (SCT) and the capillary endothelium of placental villi [58]. As a result, there is a violation of immune tolerance to the fetus, assuming α2,3-sialylation in the decidua and labyrinth, as has been observed in mice [57]. Progression of PE is believed to be associated with a general decrease in the synthesis of O-glycans carrying the sialyl-Tn antigen on such glycoproteins as mucin 1 (MUC1), CD44, integrins and osteopontin, which indirectly confirms the observed loss of MUC1 in the human placenta associated with inflammation during pregnancies with PE and chorioamnionitis [57]. In addition, the balance of placental angiogenic factors associated with endothelial glycocalyx is disturbed, which can lead to various complications and miscarriages [14]. In PE and IUGR, disturbances occur in the placental expression of genes sensitive to hypoxia, but the phenotypic manifestation of these disturbances depends on the timing and mechanism of their initiation [56]. Chronic ischemia of the placenta, as well as its ischemia-reperfusion, which develops in PE as a result of the impaired remodeling of the spiral arteries, promotes the release of antiangiogenic factors (soluble fms-like tyrosine kinase 1 (sFlt-1) and soluble endoglin (sEng)) and a decrease in proangiogenic factors (VEGF and placental growth factor (PlGF)) by the placenta, and the entry of these factors into the mother’s bloodstream, which leads to maternal endothelial dysfunction [59]. McCracken et al. (2022) revealed the overexpression of HIF-1α/HIF-2α in the placental syncytiotrophoblast layer in both PE and severe IUGR, as a result of impaired binding of HIF-1α to a protein involved in its degradation process (von Hippel–Lindau protein) in IUGR placenta, and even more so in PE [56].

Lectin analysis revealed that disruption of the expression of glycocalyx fucosylated glycans of cells within the placental barrier is associated with a change in the morphometric parameters of the villi, which can affect the function of the placental barrier [58]. The composition of the endothelial glycocalyx is normally close to that of the glycocalyx SCT. In PE, changes affect fucosylated glycans of the endothelial glycocalyx (Lewis Y (Le^y^) and H-type 2 structures) involved in the regulation of angiogenesis processes. In placentas with PE of varying severity, the expression of fucosylated glycans changed from most glycans having a Lewis X structure (presented in normal endothelial glycocalyx) to the predominance of glycans with Le^y^ and H-type 2 structures and glycans with core α1,6-fucose (α1,6-Fuc) [58]. The degree of expression of Fuc-containing glycans of the endothelial glycocalyx of the terminal villi of the placenta is dependent on the severity of PE [58].

Additionally, in human-skin microvascular endothelial cells and FUT2 null mice, fibroblast growth factor receptor 2 (FGFR2) and VEGF expression in endothelial cells was shown to be specifically dependent on fucosylation by FUT2, but not by FUT1 [60]. FUT2 mRNA expression was induced by IL-1β in a time-dependent manner, and FUT2 protein abundance was upregulated by cytokines IL-1β and TNF-α, which activate the ERK1/2 signaling pathway [60]. This induction is cell-type-dependent, as these cytokines had no such effect on a human endometrial adenocarcinoma cell line. Lack of fucosylation by FUT2 resulted in a 2-fold decrease in hemoglobin in null FUT2 mice compared with wild-type mice, as well as the dysregulation of cell migration and tube formation following basic fibroblast growth factor (bFGF) induction in vitro [60].

Another complication of pregnancy leading to oxidative stress is GDM, which results in a loss of localization of the glucose transporter 1 (GLUT1) on the SCT membrane. The incubation in vitro of primary trophoblasts from normal placentas under conditions similar to hyperglycemia causes a decrease in GLUT1 mRNA expression that is positively correlated with oxidative stress [28].

### 3.3. Intestinal Ischemia and NEC

Postnatal intestinal ischemia develops in response to the inability of the underdeveloped microvascular network to meet the intestinal oxygen demand of the metabolic processes triggered by enteral nutrition and bacterial colonization [61]. Preterm infants typically develop NEC in the second to third week of life [8]. The mechanism of initiation of NEC by enteral nutrition with artificial mixtures may be associated with the development of hypoxia as a result of insufficient postprandial intestinal hyperemia and mucosal damage. In normal adults, to meet the energy needs of digestion in the presence of oxygen, intestinal blood flow increases by almost three times compared with the initial level after a meal (postprandial hyperemia) [61].

Several studies confirmed the pathogenetic role of hypoxia in the development of NEC. Feeding-induced intestinal hypoxia in young NEC mice was associated with poor postprandial intestinal hyperemia due to inadequate microvasculature and blood flow in the immature intestine in newborn mice [61]. This effect disappeared over time as the capillary network in the intestinal villi matured. Formula-fed neonatal mice had higher HIF-1α levels in the gut than breast-fed mice, and significantly increased expression of the hypoxia marker genes *GLUT1* and HIF-prolyl hydroxylase 3 (*PHD3*) in the ileum, but not in the liver, kidneys, or heart, which indicates the tissue-specific nature of hypoxia [61]. They also had higher mRNA expression levels of the cytokines IL-6, IL-1β, IL-10, and TNF-α. Blocking NO-dependent postprandial hyperemia increases the severity of NEC, while arginine supports NO production, which helps to reduce the severity of NEC. At the same time, breastfeeding puppies did not cause intestinal damage, and did not induce an increase in the level of the hypoxia marker pimonidazole [61].

Koike et al. (2020) noted that severely damaged intestinal villus tips in the most NEC-affected terminal ileum had a high level of HIF-1α expression, but microvessels were completely absent [62]. In general, with NEC, there is a significant narrowing and a decrease in the density and length of intravillous arterioles, and a significant decrease in blood flow in the intestinal wall, according to Dopplerography [62].

### 3.4. Epithelial Barrier Dysfunction

One of the main manifestations of NEC is destruction of the intestinal mucosa, including degradation of the mucin layer and the integrity of intercellular contacts in the epithelial layer. In the feces of preterm infants, significantly lower levels of intestinal mucosal barrier proteins were detected compared with full-term infants, specifically for mucin-5AC (MUC5AC), trefoil factor 2 (TFF2), and trefoil factor 3 (TFF3), which may indicate a thinner and less stable mucus layer in the gastrointestinal tract [63].

Glycans of the epithelial cell glycocalyx and the mucin layer of the gastrointestinal tract play an important role in interactions between the host organism and colonizing microbiota. As nutrients and adhesion sites, they are factors in the selection of specific commensals, and they are also a component of the innate immune system that protects the body against adhesion of pathogenic viruses and bacteria [15]. One of the major modifications of these glycans is terminal sialylation and fucosylation, catalyzed by the respective glycosyltransferases, which can change dynamically throughout ontogeny, and are controlled by multiple factors. In the neonatal period in the epithelium of the duodenum between birth and weaning, intestinal sialylation is carried out by beta-galactosamide-alpha-2,6-sialyltransferase 1 (ST6GAL1); α2,6-sialyl glycans promote the colonization of specific *Bacillota*, mainly bacteria of the genus *Clostridiodes*, while inhibiting the colonization of *Helicobacter* and *Bilophila*. Impaired ST6GAL1 expression induces a local and systemic Th17-associated immune response and promotes epithelial hyperplasia [64]. Intensive fucosylation of ileal glycans begins at weaning with an increase in bacterial load [15]. However, it can be assumed that it also occurs less intensively in the previous period, mainly limited to the process of creating tight junctions.

### 3.5. The Role of Fucosylation in the Regulation of TLR-4-Mediated Activation of the Notch Signaling Pathway

The activation of TLR4 in the intestinal epithelium by LPS is crucial for the development of NEC, which leads to the formation of an intramembrane complex of TLR4 and CD14 and initiates an MYD88-dependent signaling pathway that activates nuclear factor kappa B (NF-κB) [11]. The triggered inflammatory cascade leads, in particular, to the release of proinflammatory cytokines including IL-6, IL-1 and TNF-α, the expression levels of which are increased in NEC [11]. This leads to the inhibition of mucosal repair by reducing enterocyte migration, which requires the activation of focal adhesion and the induction of integrins [17], and reduces the production of goblet cells, leading to the loss of the protective mucin barrier [11]. A study on the regulation of differentiation of small intestinal epithelial cells in premature mice pups revealed increased expression of TLR4 compared to full-term pups. This was associated with the role of intracellular TLR4 in activation of the Notch signaling pathway, which prevents Lgr5-positive stem cells from differentiating toward goblet cells in organogenesis during gut development [65]. This means that at during premature birth, onset of colonization by the environmental microbiota provokes TLR4 present in the still developing gut to switch from a developmental role to an inflammatory role. Endothelial TLR4 signaling in response to the entry of LPS into the circulation after mucosal injury reduces the expression of endothelial NO synthase (eNOS), and accordingly, the level of NO production, while vasoconstrictive endothelin-1 is increased in NEC [17]. Interestingly, CD14 and Notch functionality can be regulated by fucosylation. The absence of core α1,6-Fuc in CD14 results in the impaired internalization of TLR4 and CD14. The regulatory role of core α1,6-Fuc may be cell-type-specific and is thought to regulate the endothelial response to LPS [66]. It has been reported that supplementation with the major human milk oligosaccharides 2-fucosyllactose (2′-FL) and 6-sialyllactose (6′-SL), but not lactose, inhibits TLR4 activation in NEC animal models, protecting against NEC [67].

Local inflammation caused by TLR4 activation alters the intestinal epithelial barrier. Epithelial cells form tight junctions with each other due to protein complexes, including claudins and occludins, which allow them to regulate penetration of the contents of the intestinal lumen, including bacteria. Some tight junction proteins are underexpressed in preterm infants, making their epithelium more susceptible to damage during NEC [11]. A mouse model of NEC also showed that elevated intestinal permeability prior to NEC was associated with the internalization of claudin-4 and occludin. The administration of *Bifidobacterium infantis* prevented these changes and reduced the incidence of NEC [68]. This may be partly related to the fact that TLR9 activation by probiotic bacteria or bacterial DNA leads to reciprocal inhibition of TLR4 and limits the severity of NEC in mice and humans. In contrast to the increased expression of TLR4 in infants and mice with NEC, expression of TLR9 in the intestine is reduced [17].

Inadequate expression of tight junctions has been observed in preterm infants with NEC, which is a unique presentation related to the disease, and not just the developmental stage. In addition to a significant decrease in the expression of the tight junction protein genes zonula occludens-1 protein (ZO-1), occludin, cingulin, and claudin-4 in NEC, the expression of genes encoding TLR4, Bcl-2-associated X protein (BAX) and sirtuin 1 (SIRT1), and HIF-1α was upregulated in NEC patients [69]. In the blood of patients with NEC, SIRT1 was downregulated and HIF-1α was elevated. An NEC-like cell in vitro model showed that SIRT1 overexpression inhibits LPS-induced cell apoptosis and suppresses high expression of proinflammatory factors (IL-6, IL-8 and TNF-α) and decreased expression of tight junction proteins (ZO-1, ZO-2 and claudin-4). SIRT1 overexpression also suppressed HIF-1α expression and activity, while SIRT1 knockdown produced the opposite effect [70]. Högberg et al. (2013) studied the regulation of claudin and gap junction protein gene expression in hypoxia/re-oxygenation NEC model treatment, and also demonstrated that tight junction disruption may be associated with secondary hypoxia [71]. Evidence has also recently emerged demonstrating that this may be due to impaired fucosylation of tight junction proteins. Breast milk administration and probiotics are known to effectively protect against NEC [56,72], while abnormal bacterial colonization and formula feeding are major postnatal factors contributing to NEC, apart from prematurity [73]. The oligosaccharide composition of breast milk largely depends on the secretor status of the mother and GA; in secretor mothers it is dominated by fucosylated oligosaccharides [74], while formulas are based on cow milk, where they are practically absent [75]. Analysis of available data suggests that the basis of many disorders in NEC is the lack of Fuc, obtained from fucosylated glycans of glycoproteins and oligosaccharides of breast milk during their degradation by infant bifidobacteria.

## 4. Fucosylated Glycans and the Risk of Developing NEC

Although some researchers predict that the role of non-genetic risk factors in the pathogenesis of NEC is much more significant than that of genetic ones [9], recent data indicate a strong predisposition to preterm birth and the development of NEC in nonsecretor infants. These infants have allele polymorphism in FUT2 gene, resulting in the inability to synthesize H-antigen in mucins of saliva, mucus and intestinal epithelial cells [76,77,78]. Among premature infants with NEC stage II and III, a significantly higher proportion of ‘non-secretors’ was revealed (35.3% vs. 11.1%) [78]. A lower level of fucosylation of the intestinal epithelium was found in samples of the intestinal epithelium of infants with NEC [79]. Low H-antigen secretion has been associated with the risk of NEC in preterm infants, and nonsecretor phenotype has been associated with Gram-negative sepsis. Additionally, premature nonsecretor infants displayed significantly higher mortality compared with premature secretors (15% vs. 2%) [77]. By contrast, Demmert et al. (2015) did not find an association between FUT2 polymorphism in children and the risk of developing NEC. The authors noted a trend towards a higher incidence of early neonatal sepsis in individuals with AA polymorphism, partly due to the combined effect of FUT2 and premature rupture of membranes [80]. Data on the incidence of NEC in children of nonsecretor mothers are limited.

Mother’s milk contains hormones, growth factors, cytokines, chemokines and immunoglobulins, mainly IgA, lactoferrin and lysozyme, antimicrobial peptides and other regulatory proteins, microRNAs, lactose and oligosaccharides, microorganisms originating from the mother’s intestines, and their metabolic products [81,82]. Breast milk is not only a source of nutrients and an inoculum for the intestinal microbiome of the child; the biologically active compounds contained in it can perform regulatory functions, and they are often polyfunctional. For example, glycoconjugates, oligosaccharides and immunoglobulins are components of the immune system that protect the child during development of the immune system. Furthermore, glycan glycoconjugates and oligosaccharides are a source of carbohydrates for selected microorganisms of the intestinal microbiota on the one hand, an obstacle to the colonization of the intestine by pathogens on the other [83]. However, there is another aspect of their degradation by microorganisms, which is largely unexplored at present; they are a source of sugars that can be utilized by cells of the child’s body. Currently, we have separate ideas about the composition and functions of glycans, their metabolism by microorganisms, and how the oligosaccharide composition can change during lactation and thus modify the composition of the intestinal microbiota [74,84,85,86]; however, how the released bacterial sugars can participate in the ongoing development of the intestine and protect it from diseases such as NEC remains poorly understood. Nevertheless, the occurrence of such processes is very likely, since infants of nonsecretor mothers seem to have an increased risk of developing NEC, associated both with the composition of human milk oligosaccharides (HMO) and with less colonization of their intestines by bifidobacteria [87]. Additionally, the H-antigen nonsecretor status in mothers is an independent risk factor for preterm birth [88]. Several HMOs have been reported to cause growth arrest in intestinal epithelial cells, induce a shift in proliferation to differentiation, enhance the maturation of mucin-2 (MUC2)-producing goblet cells, increase the expression of intercellular tight junction proteins zona occludin and claudin-1, reduce TLR4 expression in enterocytes [89], and maintain intestinal mesenteric perfusion via preserved intestinal endothelial NO synthase expression [72].

We believe that, in addition to the above effects of HMO, exogenous L-Fuc obtained from the degradation of fucosylated HMOs may be involved in the regulation of many cellular processes that are disrupted in NEC.

GDP-L-Fuc is a glycosylation substrate for 13 different fucosyltransferases, including FUT2 [90]. Different types of fucosyltransferases also seem to be expressed differently in cells and tissues [91]. It is known that mammalian cells can use GDP-L-Fuc for the synthesis of fucosylated glycan, synthesized either via a de novo pathway, from incoming free Fuc obtained from extracellular sources, or from the salvage pathway as a result of intracellular degradation of glycoproteins and glycolipids [92]. For a long time, based on experiments on HeLa cells using isotopically labelled 0.3 μM Fuc, it was believed that the de novo pathway provides >90% GDP-L-Fuc and the salvage pathway <10%. This led most researchers to conclude that the de novo pathway is more important. However, it has recently been revealed that the degree of Fuc utilization of these pathways by a cell depends on the concentration of exogenous Fuc. The use of the de novo pathway decreases as the concentration of exogenous Fuc increases up to a complete stop at ~50 μM, while the salvage pathway does not demonstrate such a dependence on exogenous Fuc [93]. In various cell lines, it was revealed that exogenous Fuc correlates exactly to fucosylation [93,94]. It has long been believed that GDP-L- Fuc from different sources forms a common pool [92]. However, Sosicka et al. (2020) revealed that the GDP-L- Fuc pool is heterogeneous, and different types of fucosyltransferases are used for fucosylation glycans pools of different origins of GDP-L-Fuc [93]. Specifically, protein O-fucosyltransferases POFUT1 and POFUT2 catalyze the O-fucosylation of epidermal growth factor (EGF)-like repeats and thrombospondin type 1 repeats (TSR), respectively, and Notch1 O-fucosylation is catalyzed by POFUT1. While POFUT1 uses predominantly GDP-L-Fuc obtained from exogenous Fuc along with that obtained from the salvage pathway, POFUT2 uses GDP-L- Fuc predominantly from the de novo pathway. Moreover, FUT8 catalyzes the formation of the 1,6α-Fuc bond in N-glycans using primarily exogenous L-Fuc. There is a difference in the intracellular localization of different types of fucosyltransferases; hence, Sosicka et al. (2020) suggested that different fucosyltransferases receive GDP-L-Fuc from different pools [93]. This is also supported by the presence of different transporters for GDP-L-Fuc of different origins. In addition to the transporters SLC35C1 and SLC35C2, which transport GDP-L-Fuc synthesized de novo, there is an as-yet-unidentified transporter for GDP-L-Fuc derived from the salvage pathway [95]. In addition, expression of the GDP-L-Fuc transporter (SLC35C1) gene is regulated by transforming growth factor-β1 (TGF-β1) via two identical octameric GC-rich motifs in its promoter, and this is specific because TGF-β1 does not stimulate the expression of the CMP-sialic acid and UDP-GlcA/GalNAc transporters, and the TGF-β1 receptor itself is modified by fucosylation [96]. FUT2 for fucosylation in intestinal epithelial cells uses GDP-L-Fuc of both endogenous and exogenous origin [97]. This evidence suggests that there is another level of signal regulation carried out by exogenous Fuc.

A decrease in epithelial fucosylation was observed in tissue samples from patients with NEC. In mice with NEC, the level of fucosylated epithelial cells, as well as the level of FUT2 epithelial expression, was significantly lower than in control mice. A decrease in the level of fucosylation-initiating IL-22 as a result of fewer of its RORγt +ILC3 producing cells in the intestines of mice with NEC was also observed [79].

Disruption of endogenous synthesis of GDP-L-Fuc upon removal of the FX protein (GDP-4-keto-6-deoxymannose 3,5- epimerase- 4-reductase) in mice resulted in the inhibition of fucosylation of the intestinal epithelium and the development of spontaneous colitis [97]. Oral administration of L-Fuc reduced the severity of DSS-induced colitis. Research on an in vivo mouse model of dextran sulfate sodium (DSS)-induced spontaneous colitis, similar to research on an in vitro monolayer of Caco-2 cells and colonic epithelial cells, demonstrated that the administration of L-Fuc protected the integrity of the intestinal epithelial barrier. DSS significantly reduces the level of the tight junction proteins zonula occludens-1 (ZO-1) and occludin in monolayers of Caco-2 cells. However, the addition of exogenous L-Fuc to DSS-treated cells not only restored high levels of expression of the tight junction proteins ZO-1 and occludin, but also resulted in the higher expression of exogenous pathway enzymes (fucokinase (FUK), GDP-β-l-fucose pyrophosphorylase (GFPP), GDP-Fucose transporter (F-Tr)) for GDP-L-Fuc synthesis, while the expression of endogenous pathway enzymes GDP-d-mannose-4,6-dehydratase (GMD) and FX did not change [97]. Another study revealed that exogenous L-Fuc was not metabolized by differentiated Caco-2 cells, but its direct interaction with cells led to the modulation of inflammatory reactions, the regulation of the development, the proliferation of hematopoietic stem cells, and the possible restitution of intestinal epithelial cells in in vitro models [94].

## 5. Human Milk Oligosaccharides and Formation of the Intestinal Microbiome

De Leoz et al. (2012) found that the milk of mothers of term infants has a higher content of fucosylated HMOs than milk from women delivering preterm [98]. Milk for premature infants contains more protein, carbohydrates, and sodium, but the protein content decreases over time and is not insufficient for the growth of premature infants. The proportions of fucosylated and sialylated HMOs in milk of mothers of term infants are typically 60–80% and 10–15%, while milk from preterm infants contains more sialylated HMOs, and these ratios do not change significantly over time [98]. Premature infants, even when breastfed, have a low content of *Bifidobacteriaceae* and are more susceptible to intestinal infections. Reduced fucosylation of the intestinal epithelium has been noted in patients with NEC and an experimental model of NEC in mice [99]. The stable synthesis of fucosylated milk oligosaccharides was impaired in secretory mothers whose children suffered from NEC [99].

Several works demonstrate the existence of a relationship between the oligosaccharide composition of mother’s milk, her secretor status, and the composition of the fecal microbiota of infants [84,85,86]. In general, the abundance of *Lactobacillaceae*, *Enterococcus* and *Streptococcus* was lower, and *Bifidobacterium* was less common in milk samples from non-secretor mothers compared with secretors. Despite the similar diversity and richness, milk samples from nonsecretor mothers had a lower abundance of *Actinomycetota* and more *Enterobacteriaceae*, *Lactobacillaceae* and *Staphylococcaceae* [86]. The ability of individual groups of HMOs to maintain or suppress the growth of individual members of the infant microbiome was demonstrated in a 4-month study on changes in the infant gut microbiome and breast milk bacterial profile [84]. The authors reported limited ability to stratify based on maternal secretor status to analyze the association between HMO consumption and infant microbiomes, but the results are of interest as a preliminary assessment of the existence of such an association. The daily intake of various HMOs differed over time between infants of secretor and nonsecretor mothers, and the content of various bacterial operational taxonomic units (OTUs) also changed depending on HMO intake. In gut microbiome samples, 10 OTUs were identified, accounting for 62.7–77.5% of the total bacterial profile, and their relative abundances varied over time. The prevalence of OTUs attributed to *S. epidermidis*, *S. salivarius*, *Acinetobacter johnsonii*, *Veillonella nakazawae*, *Streptococcus lactarius*, *Dolosigranulum pigrum* and *Staphylococcus hominis* changed most significantly over time. In infant fecal samples, 14 OTUs were detected, collectively representing 69.4–77.9% of the total bacterial profile, and their relative abundances also varied over time. Five OTUs were assigned to *Bifidobacterium* species, namely *B. longum subsp. infantis*, *B. breve*, *B. longum* subsp. *longum*, *B. adolescentis* and *B. pseudocatenulatum*, and they dominated the bacterial profile at all time points, increasing from days 2–5 (38.0%) to 90 (59.4%), then decreasing by day 120 (44.8%). The presence-absence of OTUs related to *S. epidermidis*, *S. salivarius*, *V. nakazawae*, *Bacteroides fragilis* and *B. longum* changed significantly over time.

Interestingly, the concentrations of 13 HMOs in secretor mothers and four HMOs in non–secretor mothers decreased during the first 4 months, except for 3-fucosyllactose (3′-FL), which increased in both secretor and non-secretor mothers as lactation progressed [84]. High 3′-FL intake was associated with a lack of OTU *S. salivarius*, while higher 3-sialyllactose (3′-SL) intake was associated with a lack of OTU *B. longum* subsp. *infantis* [84]. The ability of various HMOs to support the growth of certain representatives of the infant’s intestinal microbiota is associated with the presence of appropriate bacterial enzymatic and transport systems for their utilization, which leads to the use of extracellular and intracellular HMO digestion strategies. This also explains the bacterial cross-feeding strategy [100]. A notable example is the various strains of *B. bifidum* and *B. longum*, a number of which can use extracellular glycosidases, in particular various α-fucosidases, for the degradation of fucosylated HMOs, and some have intracellular α-fucosidases [100,101]. Additionally, transporters of some strains of *Lacticaseibacillus casei* require Fuc in structure of HMOs for their recognition and transport into cells, although Fuc itself is not metabolised by bacteria [102]. Formed during the extracellular degradation of fucosylated HMOs or released into the growth medium, Fuc can serve as a source of sugars for other *Bifidobacterium* strains that do not have their own α-fucosidases. However, it can be assumed that this Fuc can be an exogenous Fuc source for epithelial cells as well. Possessing a different HMO disposal strategy, probiotic strains may explain why some infants with NEC are not protected by probiotics. Currently, we have a very crude understanding of the regulation and time windows of the development of the digestive and immune systems under the control of various metabolites in a child, including those produced by the microbiota. However, it is clear that all these processes are synchronized with the development of the intestinal microbiota.

## 6. Maternal Microbiome Dysbiosis as a Risk Factor for NEC

The maternal gut microbiota is one of the most important microbial sources for the initial colonization of the neonatal gut [103], accounting for a fifth of the contribution to the overall gut microbiome of infants [104]. Representatives of the maternal microbiome from other parts of the body, including vaginal *Lactobacillaceae*, are likely to be allochthonous microorganisms that disappear over time from the lower gastrointestinal tract of infants [104]. The mode of delivery affects the redistribution of microbial sources to the neonatal gut and may result in a reduction in maternal intestinal colonizers in the infant gut. In vaginal delivery, about 72% of neonatal early colonizers were shown to match the species found in their own mother’s stool, while in cesarean delivery only 41% of these species were found in newborns [46].

The maternal intestinal microbiota changes during pregnancy. Mothers of full-term neonates showed a significant increase in *Pseudomonadota* and *Actinomycetota* by the third trimester of pregnancy, a decrease in alpha diversity and OTU number, and an increase in beta diversity, which persisted 1 month after birth. The bacteria identified in the first trimester belonged mainly to the order *Eubacteriales* and phylum *Bacillota* (e.g., butyrate producers such as *Faecalibacterium* and *Eubacterium*), while the third trimester was dominated by members of the family *Enterobacteriaceae* and the genus *Streptococcus* [43].

The gut microbiota of mothers in the third trimester of pregnancy can induce inflammation, as has been shown by fecal microbiota transplantation in mice. This can result in increased levels of proinflammatory markers (IL-2, IL-5, IL-6, IL-1β, and lipocalin) in the cecum and stools. Furthermore, the composition of the infant intestinal microbiota was similar to the maternal composition during the first trimester of pregnancy [43].

However, according to Yang et al. (2020), in mothers of full-term infants, the composition of the intestinal microbiota did not change significantly during pregnancy [105]. Additionally, the composition of the intestinal microbiota showed individual variability depending on the mother’s somatic status (gestational diabetes, age, body mass index, weight gain during pregnancy, etc.). A limitation of this study was the lack of longitudinal data.

Maternal secretory status, as determined by genotyping of the *FUT2* gene, affected the maternal gut microbiome during pregnancy. Nonsecretor mothers showed a significant decrease in the abundances of *Clostridium coccoides*, *Ruminococcus* and members of the *Bacteroides-Prevotella* group from the first to the second trimester of pregnancy [106]. In the first trimester, nonsecretor mothers showed a significantly lower abundance of *Bifidobacterium*. The microbiome diversity in nonsecretor mothers was significantly decreased by the third trimester [106].

As the maternal microbiome is the main bacterial source for the infant gut, an aberrant postpartum microbiota in the mother may have negative health outcomes. Several studies have revealed changes in the intestinal microbiota in mothers with PE, gestational diabetes mellitus, hypertension during pregnancy, and in mothers with IUGR in neonates that correlate with a number of metabolic disorders, and lead to the development of an inflammatory process [43,107,108].

### 6.1. The Maternal Microbiome in Preterm Birth

Dysbiosis of both the vaginal and intestinal microbiota was identified in pregnant women with preterm birth. A common feature of disorders of the vaginal microbiota that precedes preterm labor and miscarriages in the first trimester of pregnancy was a low abundance of *Lactobacillaceae* [109,110,111].

The gut microbiome of mothers who gave birth prematurely was dominated by *Bacillota*, and there were few *Actinomycetota* [44,103]. These mothers had significantly fewer OTUs belonging to the genera *Bifidobacterium* and *Streptococcus*, as well as the order *Eubacteriales* [44]. The prevalence of *Bacteroidaceae* (22.5%), *Lachnospiraceae* (22.4%) and *Oscillospiraceae* (21.6%) was also noted. The impact if the mother’s gut microbiota on the preterm neonate gut microbiota was higher in spontaneous compared to induced preterm birth. GA or mode of delivery did not affect the impact of maternal to neonatal gut microbiota [103].

Analysis of the fecal microbiome in Zimbabwean women showed that *Slackia isoflavoniconvertens* was an important taxonomic predictor of longer pregnancy. The predictor of shortened pregnancy was the presence in the microbiome of *Prevotella copri* associated with more pronounced intestinal inflammation and bacterial translocation. The mother’s gut microbiome composition during pregnancy predicted neonate birth weight and weight-for-age z-score (WAZ) by 1 month of age. The most important predictors of infant weight at birth and WAZ by 1 month were bacteria involved in starch degradation and SCFA production, namely *Oscillospiraceae*, *Lachnospiraceae* and *Eubacteriaceae*, but there was variability in the data, and most effects were seen only at the extremes of the abundance distribution [112].

Data on gut microbiome diversity in mothers who underwent preterm birth are controversial. According to Hiltunen et al. (2022), mothers showed no statistical difference in alpha or beta diversity according to GA or delivery mode [103]. However, several studies revealed a significantly lower alpha diversity of the gut microbiome in mothers who delivered prematurely [44,113]. According to Dahl et al. (2017), low gut-microbiome diversity, along with altered microbial composition, may contribute to increased inflammation during pregnancy and a higher risk of preterm birth [44].

**Figure 1:**
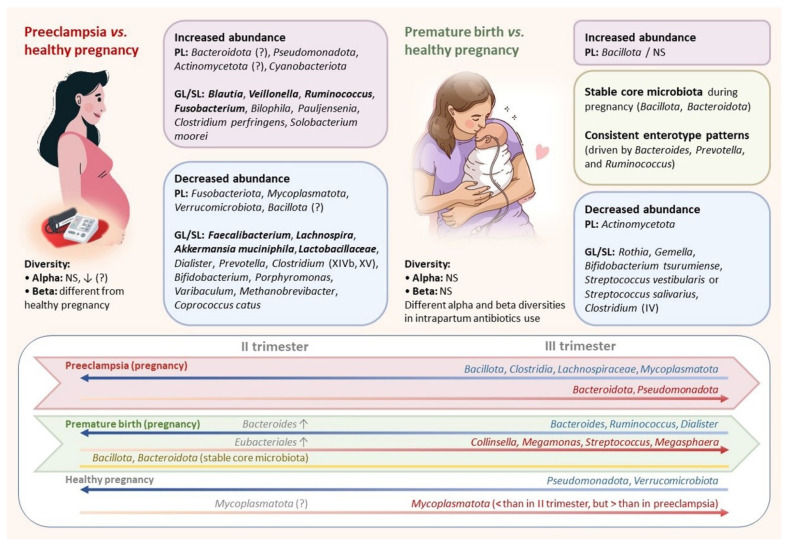
Features of maternal intestinal microbiome in preeclampsia and in premature birth. Abbreviations: PL—phylum level; GL/SL—genus level/species level; NS—not significant; (?)—research data are controversial. Taxa associated with clinical characteristics (according to research data) are in bold. This figure has been designed using assets from Freepik.com (accessed on 24 December 2022).

Antibacterial therapy during spontaneous preterm labor also affects the mother’s gut microbiome. Hiltunen et al. (2022) found that in this case, mothers in the first 3 days after birth had a higher content of *Bacillota*, *Fusobacteriota*, *Pseudomonadota* and *Actinomycetota* compared with mothers not exposed to antibiotics. Mothers who did not receive an intrapartum antibiotic had a higher *Porphyromonadaceae* abundance. Mothers who received antibiotic therapy showed a higher abundance *of Roseburia*, while mothers who did not have a higher abundance of *Macellibacteroides.* Statistically significant differences in these two groups of mothers were observed in alpha and beta diversity of the gut microbiome [103]. Features of the intestinal microbiome in mothers delivering prematurely are summarized in Figure 1. Research data of maternal gut microbiome in premature birth are presented in Table 2.

### 6.2. The Maternal Gut Microbiome in Pre-Eclampsia

Many studies have identified changes in the composition of the gut microbiome during pregnancy complicated by PE. Despite some differences in intestinal microbiome composition at a low taxonomic level in mothers with PE, a number of common features were identified in them compared to healthy mothers (Figure 1):

1. A greater abundance of *Gammaproteobacteria* in the fecal microbiota [114,115];
2. A lower abundance of *Bacillota* in the fecal microbiota [115,116];
3. A lower abundance of *Bifidobacteriaceae* [115,117];
4. Lower levels of SCFA-producing bacteria, in particular a reduced abundance of *Faecalibacterium* [108,117,118,119];
5. A lower abundance of *Mycoplasmatota* bacteria by the third trimester in mothers with PE compared to those without [107,108,117]; no difference was found in the levels of *Mycoplasmatota* [114];
6. A greater abundance of *Blautia* and *Ruminococcus* bacteria [108,117].

The microbiome during pregnancy may contribute by modulating vascular function via NO, immune mechanisms (IL-17) and vasoactive metabolites such as trimethylamine-N-oxide (TMAO) [116]. Bacterially produced SCFAs can affect host blood pressure via plasminogen activator inhibitor-1 (PAI-1) or have a direct effect on vasodilation [120]. Aromatic L-amino acid decarboxylases of intestinal bacteria (*Lactiplantibacillus plantarum*, *Lactobacillus delbrueckii* subsp. *bulgaricus* and *Enterococcus faecalis*) convert the amino acid tyrosine to tyramine, which can lead to the excessive release of norepinephrine and epinephrine from sympathetic nerve endings and adrenal medulla, respectively, contributing to the development of hypertension [59]. Huang et al. (2021) revealed a negative correlation between the abundance of *Lactobacillaceae* and blood pressure and urine protein levels in patients with PE [121].

Transplantation of the fecal microbiota of women with PE into mice induced a PE-like phenotype in mice. Mice with this phenotype had a decreased fetal and placental weight, structural changes in the placenta, reduced regulatory T cells (Tregs) and elevated levels of Th17 cells. In addition, mice had gut barrier dysfunction manifested by decreased expression of tight junction proteins ZO-1 and ZO-2, claudin-4, and occludin in the colon, and mild inflammatory cell infiltration in the colon [118].

Gut microbiome features in women with PE persisted for 6 weeks postpartum [108], and, therefore, could influence the formation of the infant’s intestinal microbiome. Neonates born to mothers with hypertension during pregnancy had significantly lower alpha diversity in the gut microbiome at 6 months of age than newborns of mothers without hypertension [122]. The gut microbiome composition of infants born to hypertensive mothers differed by a greater abundance of the phylum *Bacteroidota*, classes *Betaproteobacteria*, *Coriobacteriia* and *Bacteroidia*, orders *Bacteroidales*, *Burkholderiales* and *Coriobacteriales*, families *Alcaligenaceae* and *Coriobacteriaceae*, *Sutterella*, *Clostridium aldenense* and *Bacteroides* compared to neonates of mothers without hypertension. *Streptococcus infantis* levels in children from mothers with hypertension were significantly higher [122].

Research data of maternal gut microbiome in pre-eclampsia (PE) and pregnancy-induced hypertension (PIH) are presented in Table 3.

### 6.3. The Gut Microbiome of Mothers and Neonates with IUGR

Data on the gut microbiome composition of neonates with IUGR and their mothers are limited. Tu et al. (2022) revealed a greater abundance of the phylum *Bacillota*, and the genera *Bacteroides*, *Faecalibacterium*, and *Lachnospira* (of the *Lachnospiraceae* family) in the mothers of full-term IUGR infants [127].

Yang et al. (2022) studied the relative effects of intrauterine environment factors on the neonatal-intestinal-microbiome development of discordant and concordant monochorionic (MCDA) and dichorionic twins (DCDA) [128]. Infants with IUGR from discordant MCDA twins showed a significantly lower abundance of *Enterococcus*, especially *E. faecium*, and greater levels of *Coprococcus* and *Oscillospira. The* increased abundance of butyrate-producing bacteria in neonates with IUGR may be a mechanism to compensate for intrauterine ‘malnutrition’ by regulating the energy metabolism of the intestinal epithelium and reducing inflammatory reactions [128].

Discordant MCDA twins simultaneously showing a low abundance of *E. faecium* had low levels of methionine and cysteine in fecal samples, which counteract oxidative stress and are involved in DNA methylation and fetal growth [128].

Intrauterine hypoxia may also influence the composition of the gut microbiome after birth. One experimental study revealed a greater level of *Bacillota* in the gut microbiome of rats who underwent intrauterine hypoxia compared to controls, in which *Pseudomonadota* predominated [129]. Rats that underwent intrauterine hypoxia had a greater relative abundance of *Bacillota* and *Bacteroidota*, and a higher ratio of *Bacillota* to *Bacteroidota* on day 7 of life compared to day 1 of life and the control group [129].

## 7. The Microbiome of Preterm Infants

The first few days of life are crucial for the correct development and modulation of the human gut microbiota [130]. 

Normally, the intestine of full-term infants is usually better supplied with oxygen; therefore, during the first week of life, it is mainly composed of facultative anaerobes *(Enterobacteriaceae*, *Enterococcus*, and *Streptococcus*). Facultative anaerobes consume oxygen and reduce intestinal oxygenation, which contributes to the replacement by strict anaerobes (predominantly *Bifidobacterium* and lactic acid bacteria, whose growth is supported by glycoprotein glycans and HMOs) [84]. However, preterm neonates are exposed to another environment after birth, so they develop differently from full-term infants [131].

Significant differences in gut microbiota composition between preterm and term newborns have been revealed even in meconium [132]. The meconium microbiome of preterm infants is dominated by *Bacillota* and *Bacteroidota* at the type level, and has lower alpha and beta diversity than in full-term infants. In addition, proinflammatory and metabolic effects of preterm meconium have been identified. Transplantation of the meconium microbiota from preterm infants (GA < 32 weeks) into mice was associated with significantly lower weight gain, lower plasma insulin and leptin levels, and higher expression of IL-6 mRNA in the terminal ileum compared to mice transplanted with meconium from term neonates [132]. The meconium of premature neonates has higher levels of proinflammatory markers such as neutrophil elastase and myeloperoxidase and fecal calprotectin compared to full-term meconium [133]. Heida et al. (2016) identified NEC-associated gut microbiota (*Clostridium perfringens* and *Bacteroides dorei (Phocaeicola dorei)*) in the meconium of preterm neonates with a GA < 30 weeks [134].

A study on the contribution of the mother’s gut microbiome to the initial colonization of the preterm infant’s gut found that the most abundant bacterial genus in the meconium was *Bifidobacterium*, which was also frequently present in mothers. *Bacteroides* was the most common bacterial genus in maternal stool samples, but not in meconium samples. Other dominant genera in maternal stool samples (*Blautia*, *Faecalibacterium*, and *Subdoligranulum*) were present in some meconium samples, but at a lower prevalence and abundance [135].

Zwittink et al. (2017) suggested that neonatal respiratory support may prevent colonization of the intestine by anaerobic microorganisms because it helps to maintain oxygenation in the gastrointestinal tract [41].

The mode of delivery also influences the gut microbiome formation of preterm newborns. The predominance of *Staphylococcus* in stool samples during the first three weeks of life was associated with delivery by caesarean section, while *Escherichia coli* and *Bacteroides* abundance was associated with vaginal delivery [47].

According to Heida et al. (2021), current absolute weight is a better marker of infant-gut-microbiome maturation than postconceptional age, as it is less affected by various infant-specific factors [47]. Fetal growth restriction/low weight at birth affects immune-response modulation, Paneth-cell maturation, and mucus production, which may cause weight-dependent differences in microbiome maturation [47]. Body weight at 2–4 weeks of age significantly correlated with the prevalence of *Staphylococcus* and *Enterobacteriaceae*, which was not associated with postconceptual age. Neonatal weight, irrespective of the sampling week, was likely associated with a shift in dominance in the gut microbiota from *Staphylococcus* to *Enterobacteriaceae*. Meanwhile, body weight increased, *Staphylococcus* abundance decreased, and *Enterobacteriaceae* increased [47].

In preterm infants, a higher birth weight correlated with greater levels of bifidobacterial proteins in postnatal weeks 3 to 6 [63]. Extremely preterm infants, characterized by a low mean relative content of bifidobacterial-derived proteins, had greater levels of oxidative-stress proteins compared to term infants at the second to fourth postnatal weeks (*p* ≤ 0.01). Given the high abundance of facultative anaerobes (*Enterococcus*, *Escherichia,* and *Klebsiella*) and the low abundance of *Bifidobacterium* in preterm stool samples, oxidative-stress proteins may provide facultative anaerobes with a competitive advantage [63].

Dynamic changes in the composition of the gut microbiome are different for each preterm neonate and vary depending on the clinical events that occur during treatment in the intensive care unit. Antibiotics remain the most commonly prescribed drugs in neonatal intensive care units due to the high risk of infection in newborns [48], which influences the formation of their intestinal microbiota. The effect of the antibiotics used on the microbial community largely depends on their type. However, most often, their exposure leads to the predominance of facultative anaerobes, such as *Enterococcus*, in the gut microbiome of preterm infants [136,137,138]. The alpha and beta diversity in newborns treated with antibiotics 48 h after birth did not differ from those not treated at any individual-adjusted GA [48]. The diversity depended on which taxa dominated the community. Richness and diversity were higher in mixed and *Bifidobacterium*-abundant communities compared to those with *Enterococcus*, *Streptococcus*, or *Staphylococcus* dominance [138].

The influence of the enteral nutrition substrate on the intestinal microbiome composition of preterm infants also cannot be underestimated. Neonates fed exclusively or partially breast milk at 32 weeks of gestation tended to have greater intestinal microbiome diversity [48], and infants who did not receive enteral feeding at an adjusted GA of 36 weeks had a significantly lower Shannon diversity index [48].

### Dynamics of Gut Microbial Colonization in Neonates

The process of gut microbial colonization of preterm infants goes through certain stages. According to La Rosa et al. (2014), Gram-positive Bacilli prevailed up to 8 days of life, but were soon replaced by Gram-negative facultative anaerobes (*Gammaproteobacteria*), and for 10 days in the postconceptual-age interval of 33–36 weeks, *Clostridia* were the most abundant [139]. Moles et al. (2013) revealed the prevalence of *Bacilli* in the meconium of preterm infants, while by the 3rd week of life, *Enterococcus*, *E. coli*, *Klebsiella pneumoniae*, and *Yersinia* were increased [140].

At 1 day of life, there was a decrease in *Bacilli* and an increase in *Clostridia* in each subgroup of GA [139]. Antibiotic use was associated with an increase in *Gammaproteobacteria* (for infants born after GA ≥ 26 weeks), and a decrease in *Clostridia* in stools (for infants born after GA ≤ 28 weeks). Exogenous factors did not change the overall trend in population evolution, but only its pace; the lower the GA of neonates, the lower the rate of colonization [139].

Premature infants are characterized by a predominance of facultative anaerobes and delayed colonization by obligate anaerobes such as *Bifidobacterium* [41]. The colonization process of *Bifidobacterium* appears to be delayed in preterm infants compared to term infants due to the lower frequency of breastfeeding compared to term infants [141].

Analysis of the fecal proteome showed a low bacterial load until the second postnatal week in all preterm infants [41]. In extremely preterm infants (25–27 weeks), facultative anaerobes predominated after 6 weeks of life, while in preterm infants with a GA of 30 weeks, *Bifidobacterium* predominated from postnatal week 3 [41].

The process of gut barrier maturation is closely related to the development of the gut microbial community. The intestinal barrier is permeable at birth, but within 7–10 days of life, most preterm infants experience maturation of the intestinal barrier [42]. According to Lemme-Dumit et al. (2022), slower maturation of the gut microbiota of preterm infants is associated with persistently increased intestinal permeability, a lower frequency of breastfeeding, a lower GA and body weight, and inadequate cytokine profiles [42]. Arboleya et al. (2022) revealed higher levels of systemic inflammation and increased overall permeability of the gastrointestinal tract of preterm infants, which persisted even beyond 3 weeks of age [131]. Transplantation of the early microbiota (<2 weeks of life) of a preterm infant (GA of 27 weeks) with normal weight gain (>10 g/kg/d) in gnotobiotic mice caused an increase in the height of the villi and the depth of the crypts. Cell proliferation as well as the number of goblet cells, Paneth cells, and tight junctions increased compared to changes induced by the early microbiota of a premature infant with poor weight gain (<10 g/kg/day); the changes were comparable to germ-free mice [142]. The main oligosaccharides of breast milk such as 3′-SL and 6′-SL led to a decrease in the proliferation of intestinal epithelial cells and an increase in their differentiation in vitro [72].

The authors distinguished various phenotypes [42], clusters [143], and archetypal groups [130] of the fecal microbiota of preterm infants.

Lemme-Dumit et al. (2022) identified three distinct gut microbiome phenotypes in preterm infants at 7–10 days of age [42]:

- Type S (with a prevalence of *Staphylococcus epidermidis*);
- Type E (with a predominance of *Enterobacteriaceae*—*Klebsiella pneumoniae* or *Escherichia coli*);
- Type O (wide-spectrum anaerobic and optional microorganisms—*Bifidobacterium*, *Lactobacillales*, *Veillonellales*, and *Eubacteriales*).

Types S and E were identified in preterm infants with a lower GA (<28 weeks) and body weight (<1500 g) and were associated with greater permeability of the intestinal barrier, indicating slower colonization of the intestine (because *S. epidermidis* and *Enterobacteriaceae* are the ‘first colonizers’, and anaerobic microorganisms are their successors). Type O was associated with lower intestinal permeability, later gestation (≥28 weeks), a higher body weight (≥1500 g), and breast-milk feeding.

Both S and E microbiota types, especially the *S. epidermidis*-dominated type, had a highly diverse immunological profile with great interindividual variability. Type O was distinguished by a high level of similarity within the cluster based on cytokine and chemokine profiles. Thus, while the gut microbiota develops, mucosal immunity in preterm infants becomes more stable [42].

Ho et al. (2018) revealed the dichotomy in the intestinal microbiome of preterm newborns with very low birth weight (VLBW) (GA of 27.9 ± 2.2 weeks) [143]. Two different subgroups of neonates were identified: cluster 1 was characterized initially (at an age of fewer than 2 weeks) by a low number of *Gammaproteobacteria*, which increased by 3 and 4 weeks of life; cluster 2 started with a high abundance of *Gammaproteobacteria*, which decreased by the 3rd week of life and recovered by the 4th week. The predominance of *Gammaproteobacteria* in the initial microbiome (less than 2 weeks of age) was associated with vaginal birth and antenatal steroids. Neonates of the first cluster had a lower birth weight and a lower vaginal-birth frequency [143].

Tarracchini et al. (2021) identified five archetypical subgroups named preterm community state types (PT-CST) for the fecal microbiota of preterm infants [130]. The dominant PT-CST species (*Streptococcus agalactiae*, *E. coli*, *E. faecalis*, and *K. pneumoniae*) were involved in relationships with minority members of the gut microbiota of preterm infants (*Streptococcus*, *Cutibacterium*, *Enterobacter*, *and Corynebacterium*, as well as members of the *Actinomycetota*). Minor members of the bacterial population may shape the infant-gut-microbiota community, playing a critical role in maintaining the balance of the interaction network. Archetypal groups were unevenly distributed in children with and without NEC [130].

- PT-CST1 (Enterococcus faecalis predominance, Actinomyces, Schaalia);
- PT-CST2 (Escherichia coli predominance, Bacteroides fragilis);
- PT-CST3 (Streptococcus agalacticae predominance, Streptococcus vestibularis);
- PT-CST4 (Staphylococcus epidermidis predominance);
- PT-CST5 (Klebsiella pneumoniae predominance, Akkermansia).

Thus, multiple factors affect the initial colonization of the intestines of preterm infants (GA, birth weight, mode of delivery, immaturity of the intestinal barrier, composition of the mother’s intestinal microbiome, and breast milk/formula at the start of enteral feeding) and dynamic colonization (enteral nutrition substrate, use of antibiotic therapy, infection, and prolonged stay in the neonatal intensive care unit).

## 8. Features of the Intestinal Microbiota and Metabolome in NEC

The risk of NEC is probably determined by a complex change in the composition and diversity of the microbiome, including altered interactions between dominant and minor microorganisms, since it is rarely possible to identify the predominance of a particular microorganism [130]. Wandro et al. (2018) noted that the composition of the fecal microbiome of preterm infants is unique to each infant and independent of clinical status [136]. Neonates with NEC showed a significant decrease in microbiota diversity within PT-CST, with an overgrowth of PT-CST-specific opportunistic bacteria such as *E. faecalis*, *E. coli*, *S. epidermidis*, *Clostridioides difficile*, *Ureaplasma parvum*, *Pseudomonas aeruginosa*, *Pseudomonas nosocomialis*, and *Klebsiella.* At the same time, intestinal commensals such as *Actinomyces*, *Schaalia*, *Veillonella*, *Bacteroides*, and *Streptococcus* were reduced or absent in fecal samples from patients with NEC [130].

For some predominant bacteria, a change in metabolic pathways may be possible due to the lack of necessary nutrients and the participants utilizing them. Such bacterial members, possessing the enzymes necessary under the new conditions, will have an advantage. The immature microbiome of preterm infants contributes to changes in metabolism, resulting in a metabolic state similar to fasting despite adequate caloric intake [11].

The driving force of bacterial-population shaping may be metabolic competition. Bacterial metabolism in vivo is an essential aspect of virulence [144]. Bacteria populate different niches within the host organism during infection and must adapt to take advantage of alternative nutrient sources. Interference competition occurs between several bacterial pathogens, whereby bacteria produce substances (secondary metabolites) to suppress the growth of others or actively restrict or remove a nutrient from its competitors [145]. Metabolic competition between bacteria involves several mechanisms, including the inability to utilize one substrate (the need for different metabolic niches), and different rates of substrate uptake/different hierarchies of energy use [145].

The shaping of the gut microbiota from a variety of commensal and beneficial bacterial species is essential for the host organism, as it provides competition with pathogenic bacteria [146,147]. This intestinal symbiosis also plays a key role in nutrient digestion and metabolism, vitamin synthesis, immune tolerance, intestinal mucosal maturation, and brain development [146]. Dobbler et al. (2017) found that the community of obligate anaerobes was highly influential in the intestine, had a protective role in neonates without NEC during the first four days of life, and controlled the proliferation of *Enterobacteriaceae* [148].

### 8.1. The Role of Gammaproteobacteria

Many studies have demonstrated the etiological role of *Enterobacteriaceae* in the development of NEC [130,148,149,150,151]. The predominance of *Gammaproteobacteria* and the paucity of strictly anaerobic bacteria (especially *Negativicutes*) preceded NEC in VLBW neonates [152]. According to a systematic review and meta-analysis by Pammi et al. (2017), microbial dysbiosis before NEC onset in preterm infants is characterized by the increased relative abundance of *Pseudomonadota* and decreased *Bacillota* and *Bacteroidota* [153]. Olm et al. (2019) revealed the etiological role of *Enterobacteriaceae*, in particular the *K. pneumoniae* 242_2 strain, in the development of NEC in preterm infants (detected in 52% of samples before NEC compared to 23% without NEC) [151]. Bacterial genes in the pre-NEC samples were enriched with a cluster of fimbriae 49, gene clusters of secondary metabolites (bacteriocins, sactipeptides, and butyrolactones). Ward et al. (2016) identified uropathogenic *E. coli* as a significant risk factor for NEC [154].

Oxidative stress in NEC affects not only neonatal intestinal tissue, but also the intestinal microbiota. Glutathione is a component of the antioxidant system—not only in eukaryotic cells; it can also be synthesized under oxidative stress by members of *Pseudomonadota* and a limited number of Gram-positive bacteria (some species of *Streptococcus* and *Staphylococcus aureus*, but not *Clostridium* and *Bacillus*) [155]. This gives them an advantage over groups of glutathione non-synthesizing bacteria and may be one of the explanations for the increase in *Gammaproteobacteria* abundance in NEC.

### 8.2. The Role of Clostridia

Another possible mechanism by which the gut microbiota contributes to the development of NEC is a decrease in butyric acid synthesis in the intestine under the predominance of *Pseudomonadota* and a decrease in *Bacillota*, which leads to a decrease in regulatory T cells [149]. However, the role of butyrate in the pathogenesis of NEC is controversial. Several studies have confirmed the etiological role of butyrate-producing *Clostridium sensu stricto I* in the development of NEC in preterm infants [156,157,158]. Butyrate likely contributes to the development of NEC through its ability to inhibit the proliferation of intestinal stem cells located at the base of intestinal crypts [159]. The opposite effect of butyrate on the proliferation of differentiated and undifferentiated colonocytes is known as the ‘butyrate paradox’ [160]. Heida et al. (2016) revealed an abundance of *Clostridium perfringens* (8.4%) and *Bacteroides dorei (Phocaeicola dorei)* (0.9%) in the meconium of preterm infants who subsequently developed NEC [134].

### 8.3. Is the Predominance of Gammaproteobacteria or Clostridia Dependent on GA?

The pattern of gut microbial colonization in preterm infants may differ depending on the timing of NEC onset [161]. *Clostridium sensu stricto* predominated before the early onset of NEC (≤22 days of life). Neonates with a late onset (>22 days of age) tended to have increased *Escherichia/Shigella* 6 days before the onset of NEC. The abundance of *Cronobacter* (*Gammaproteobacteria*) was also significantly higher in cases of late-onset NEC than in the control group 1–3 days before the onset of NEC [161].

Is the direction of microbiome colonization (*Clostridia*-dominated/*Gammaproteobacteria*-dominated) related to GA in preterm infants? Warner et al. revealed differences in gut microbiome composition depending on GA at birth. Infants with a GA < 27 weeks who developed NEC had a significant increase in *Gammaproteobacteria* over time, and a decrease in *Negativicutes* and the combined *Clostridia*–*Negativicutes* class. However, infants without NEC with a GA > 27 weeks showed an increase in the combined *Clostridia*–*Negativicutes* class [152].

This dependence of colonization of the gut microbiome on GA was also shown in other studies. Phenotypes of the gut microbiota S phenotype (with *Staphylococcus epidermidis* predominance) and E phenotype (with *Enterobacteriaceae* predominance) were revealed in preterm infants with a lower GA (<28 weeks) and body weight (<1500 g), and were associated with a greater permeability of the intestinal barrier and slower colonization [42]. The O phenotype (dominated by *Bifidobacterium*, *Lactobacillales*, *Veillonellales* and *Eubacteriales*) was associated with lower intestinal permeability, a later GA (≥ 28 weeks), a higher body weight (≥1500 g), and breast-milk feeding [42].

According to La Rosa et al. (2014), the primary colonizers (*Bacilli*) were replaced by *Gammaproteobacteria* shortly after birth in preterm neonates without NEC, followed by *Clostridia* predominance by 33–36 weeks postconceptual age [139]. The authors emphasized that the increase in *Clostridia* abundance occurred more gradually in infants with a lower GA at birth [139]. Moles et al. (2013) revealed the replacement of *Bacilli* by *Enterococcus*, *Escherichia coli*, *Klebsiella pneumoniae*, and *Yersinia* by the 3rd week of life in infants born at a GA ≤32 weeks [140].

It is interesting that, in term infants, the presence of *Clostridia* in the gut microbiome was noted only by the age of 4 months and increased by 12 months, as a result of weaning from breastfeeding and the transition to a more adult-like intestinal environment associated with the increased functional ability to degrade carbohydrates [46].

### 8.4. The Role of Environmental and Nosocomial Microorganisms

It is impossible to exclude the role of environmental microorganisms in the development of NEC in preterm infants. Infants who subsequently developed NEC had environmental bacteria in stool samples within the first 48 h of life, including *Dysgonomonas* (found on surfaces), *Hyphomicrobiales*, *Ralstonia*, and *Pelomonas* (found in water sources and hospital ventilators) [162]. The causative agents of nosocomial infections can also contribute to NEC in preterm infants (*K. pneumoniae*, *Proteus mirabilis*, *C. perfringens*, *Clostridium neonatale*, *Pantoea diversa*, and *S. aureus*) [130].

### 8.5. Intestinal Virome in NEC

A fundamentally new approach to the study of NEC etiology is the analysis of the intestinal virome (especially the phageome) and viral–bacterial interactions in preterm infants. Kaelin et al. (2022) revealed a decrease in viral beta diversity 10 days before the onset of NEC, while a bacterial shift was observed 25 days prior to NEC onset [163]. This was accompanied by specific viral–bacterial interactions. Most of the virome (84.5%) was unclassified viruses. The gut virome of preterm infants had high interindividual and intra-individual variability; 137 contigs associated with the development of NEC were identified and correlated with specific genera of bacteria in infants. Several contigs associated with NEC were positively correlated with *Escherichia* and *Streptococcus*, while many of the contigs at more than 10 days before NEC were negatively correlated with these genera. Correlations between NEC-associated contigs, and *Proteus* and *Bifidobacterium*, were mainly negative. On the other hand, correlations with *Acinetobacter*, *Clostridium*, *Lactobacillaceae*, and *Haemophilus* were generally positive. These specific interactions were absent in children without NEC. Of the NEC-associated contigs with at least five open reading frames, 31.7% were temperate bacteriophages and 68.3% were lytic [163].

Significantly, differences in the virome composition and gut microbiome metabolites in very-preterm neonates, but not in gut bacterial populations, can still be found at prepubertal age (5–11 years) [164]. Children aged 5–11 years born very preterm showed significantly decreased gut bacteriophage richness and significant changes in plasma and fecal metabolites. The authors speculated that the observed very-preterm-specific changes may impact the ability of the children’s microbiota to respond to various environmental changes [164].

A multi-omics study in an animal model of NEC showed that some gut bacteria (*Enterococcus* and *Subdoligranulum*) may benefit NEC by influencing bacterial phages [165]. In addition, given the data on the increased replication of *Enterobacteriaceae*, and in particular *K. pneumoniae*, in preterm infants who subsequently developed NEC [151], it is possible that *K. pneumoniae*-targeting phage therapy may, in the future, be used to prevent and treat NEC, as considered for IBD [166].

### 8.6. Gut Microbiome Metabolic Effects and Microbial Metabolites in NEC in Preterm Infants

The metabolic effects of the gut microbiome also probably play a role in the development of NEC. Multiomics studies highlight the importance of researching not only taxonomic changes in NEC, but also microbial metabolic potential, providing evidence for a microbiome–metabolome association [167]. Fu et al. (2021) revealed the involvement of microbial pathways associated with the breakdown of L-tryptophan and aromatic compounds in infants with NEC, which also remained active for 48 h after diagnosis of NEC [162]. The species composition of the microbiota was mainly represented by *Ralstonia* [162]. Metagenomic data analysis of infant fecal samples showed that genes encoding tryptophanase and indolepyruvate decarboxylase were more highly expressed in the bacteria of NEC stool samples [130]. NEC microbiomes of preterm infants (unlike preterm infants without NEC) were almost completely lacking α-fucosidase and sialidase genes. These enzymes are required for the release of L-Fuc and sialic acid from host glycans such as intestinal mucins and HMOs. The enzyme lactate dehydrogenase was overabundant in preterm infants prior to NEC development compared with their healthy peers and neonates with NEC [130]. An imbalance between lactate-producing and lactate-utilizing bacteria can lead to gastrointestinal DL-lactate accumulation in infants who later develop NEC. Thus, the level of DL-lactate, as well as a decrease in enzymes capable of utilizing HMOs, may be useful as a potential biomarker for the early diagnosis of NEC [130].

It is possible that metabolomic changes in some amino acids, such as alanine, arginine, citrulline, glutamine, histidine, phenylalanine, and proline, as well as amino acid ratios characteristic of infants with NEC, may be associated with impaired bacterial metabolism and the number of specific bacteria [167]. For example, the ratio of urinary alanine to histidine (a potential metabolite biomarker) was significantly higher in NEC and was inversely associated with the relative abundance of fecal *Propionibacterium* [168]. Thomaidou et al. (2019) showed significant alternations in the urine metabolome in preterm infants with NEC compared to controls [169]. Some of the discriminant urine metabolites (phenylalanine, tyrosine, hippuric acid, pyridoxine, riboflavin, etc.) may be associated with intestinal bacterial metabolism and could potentially be used as diagnostic biomarkers of NEC [169].

According to Stewart et al. (2016), four of the five metabolites most associated with NEC were involved in linoleate metabolism, which is potentially mediated by the microbiome [170]. The most discriminatory metabolites in NEC samples at the time of diagnosis were significantly elevated compared to controls. Furthermore, their intensity increased before the diagnosis of NEC and decreased after diagnosis, which suggests that such metabolites can potentially serve as predictive biomarkers. The authors suggested that progression toward NEC may be detected between 1–2 weeks before the current clinical diagnosis [170]. The identification of reliable NEC candidate biomarkers requires a systematic approach based on metabolomics and metagenomics, and can potentially be used both for the early diagnosis of the disease and for predicting the risk of adverse clinical outcomes much earlier than clinical manifestations [167]. Research data of the intestinal microbiome in preterm neonates with NEC are presented in Table 4.

## 9. Prediction of NEC

Considering the multifactorial nature of the causes of NEC development, developing prognostic NEC models based on multiomics approaches could be advantageous. Currently, several approaches have been proposed to predict the development of NEC, including using machine learning [34,173,174,175].

Lin et al. (2022) [176], using intestinal-microbiome data by Warner et al. (2016) [152] and Olm et al. (2019) [151], created an application for dynamic individual-NEC-risk assessments, noting the importance of *Clostridia* and *Actinomycetota* members for NEC prediction, in addition to *Bacilli* and *Gammaproteobacteria*.

The role of modifiable factors that reduce the risk of NEC should not be underestimated; the antenatal administration of corticosteroids, the prenatal education of mothers about the importance of breastfeeding, support for lactation and initiation of breast milk expression as early as possible, limiting the excessive use of antibiotics in neonates, early ‘irrigation’ of the oral cavity with colostrum (colostrum swabbing to provide oral immune therapy), and the standardization of enteral nutrition approaches may all be of relevance [177,178]. In addition, factors reducing the risk of NEC include limiting the use of H2 blockers, preventing severe anemia, preventing bacterial contamination of the feeding substrate and feeding devices, and using the kangaroo method [177]. The use of probiotics and a combination of types of probiotics also reduces the risk of NEC [178].

Chandran et al. (2021) reduced the risk of NEC in one department from 7% to 0% within four years when using methods that reduce the risk of NEC (standardization of the protocol for enteral nutrition, oropharyngeal administration of colostrum, the use of probiotics, limiting the use of H2 blockers, antibiotics, the dilution of oral drugs to limit osmolarity, and encouraging mothers to breastfeed) [179].

## 10. Remote Ischemic Conditioning (RIC) as a Therapy for NEC

Therapy for NEC is non-specific; examples include nil per os, total parenteral nutrition, and gastric decompression and antibacterial therapy [6,8,180]. According to a systematic review by Gill et al. (2022), no optimal antibiotic therapy regimen for NEC stages II and III has been identified [181]. The choice of therapy approach is made difficult due to the wide range of NEC-like diseases, such as spontaneous intestinal perforation, ischemic intestinal necrosis, enterocolitis associated with food protein intolerance, and congenital malformations of the gastrointestinal tract [180]. Given the major role of impaired microcirculation in the intestinal wall in the pathogenesis of NEC, Koike et al. (2020) suggested RIC for treating NEC [62]. The goal of RIC is to improve collateral circulation in distant target tissues susceptible to ischemia. The mechanism of RIC is based on the release of endogenous vasodilators, namely NO and hydrogen sulfide (H_2_S), which promote the vasodilation of the intestinal microvasculature, maintaining intestinal perfusion and reducing mucosal hypoxia [62].

According to an experimental study in newborn mice, RIC in the early stages of NEC (stages 1 and 2 RIC) increased blood flow velocity in the intestinal wall and improved the survival of mice with NEC. However, RIC at stage 3 did not provide significant protection against intestinal damage and did not improve survival [62]. According to Koike et al. (2020), RIC was able to prevent the disruption of intestinal wall microcirculation in response to enteral feeding in the early neonatal period [62].

## 11. Conclusions

NEC is a multifactorial disease of premature neonates, including pathogenesis contributed by alteration of intestinal microbiome development, episodes of intestinal hypoxia caused by immature intestinal wall receptors of preterm infants, and immaturity of the microvascular bed. Recent studies focused on the maternal-gut-microbiome composition, including physiological aspects [43], and complicated pregnancy (premature birth [44,103,105,113], pre-eclampsia [107,108,114,117,118,119,121], and fetal IUGR [127]) and its contribution to the initial gut colonization of preterm infants. It is currently assumed that changes in the maternal gut microbiome during pregnancy progress through certain stages during and following delivery [43]; however, some studies refute this concept [105]. The role of the maternal gut microbiome in shaping the risk of NEC is unclear and needs to be further studied. The gut microbiome of mothers of preterm infants with NEC is currently not well understood.

The influence of several factors on the gut microbiome colonization of preterm infants (mode of delivery, the maternal microbiome composition, enteral nutrition, the duration of stay in the intensive care unit, and the use of antibiotic therapy) makes research on gut microbiome composition and the identification of NEC-related pathogens particularly challenging. The microbiome of preterm infants is a unique system of dominant and commensal microorganisms, the disruption of which may contribute to the risk of NEC [130,147]. Many studies have highlighted the role of *Gammaproteobacteria* prevalence in the development of NEC [130,148,149,150,151,152,153], while other studies point to *Clostridia* as a pathogen [134,156,157,158].

Due to the nonspecificity of the early manifestations of NEC and the rapidity of its course, the search for noninvasive markers of NEC and the identification of risk factors are ongoing. There is increasing interest in studying the role of fucosylated glycans, the influence of maternal secretory status on the risk of preterm birth [88], and the effect of neonatal secretory status on the risk of NEC [77,80]. However, the influence of maternal secretory status on the risk of NEC in neonates has not yet been studied.

Fucosylated glycans on the surface of the intestinal mucosa and breast milk oligosaccharides may also be involved in gut microbiome shaping in infants [84,85,87] as well as the increased expression of intercellular tight junction proteins ZO-1 and claudin-1 [89] and the inhibition of TLR4 on enterocytes [67]. In addition, TLR4-mediated activation of the Notch signaling pathway may also be regulated by fucosylation [66].

Future research on maternal microbiome composition, the discovery of candidate biomarkers, and microbiome-based therapies will contribute to reducing the risk of NEC and NEC-related complications and improve survival. The use of fucosylated oligosaccharides [182,183] in formula and probiotics [184] could offer promising approaches to NEC prevention when breast milk is unavailable since the composition of the microbiota is largely maintained by HMOs [185].

## Figures and Tables

**Table 1:** Groups of risk factors of necrotizing enterocolitis.

Somatic Factors	Factors Related to Nutrition
Lower birth weight [22,23,27]	Formula feeding [36]
Gestational age at birth	Breast milk fortification [35]
Intrauterine growth retardation [20,29]	
Genetic predisposition [9]	
**Factors associated with hypoxia**	**Factors associated with tissue perfusion**
Persistent ductus arteriosus [22]	Arterial hypotension [27]
Red-blood-cell transfusion [35]	Hematocrit > 49.65% and mean corpuscular volume > 114.35 fl [35]
Apgar score < 7 at 5 min [22] Apgar score < 6 for 1 min, <7 for 5 min, and <8 for 10 min increases the risk of mortality in NEC [37]	Congenital heart disease [33]
Resuscitation in the delivery room	Persistent ductus arteriosus [24]
Assisted ventilation [27]	Pre-eclampsia (?)
Lower target oxygen saturation [38]	Abnormal blood flow in the umbilical artery prenatally [20,21]
Isoimmunization [22]	Placental abruption [24]
Intracranial hemorrhage ≥ Grade II [35]	
Higher blood lactate level [37]	
**Factors associated with infection**	**Gut-microbiota-related factors**
Maternal chorioamnionitis [20]	Long-term antibiotic therapy in children [39,40] Impaired intestinal colonization in the early neonatal period [41,42]
Premature rupture of membranes (?)	Features of the intestinal microbiota of the mother depending on the duration of pregnancy [43,44]
Sepsis [33,36,45]	Disruption of gut microbial colonization due to prematurity, birth by caesarean section [46,47], or formula feeding [48]
Bacterial infection [22,34]	Administration of H2 blockers that suppress acidity [34]

NEC: necrotizing enterocolitis; (?): research data on the role as a risk factor of NEC are controversial.

**Table 2:** Features of the intestinal microbiome in mothers delivering prematurely.

№	Authors, PMID/doi	Setting and Time Period	Participants and Study Design	Methods	Features of Microbiome
1	Dahl C. (2017) [44] PMID: 29069100	The Norwegian Microbiota Study (NoMIC) County hospital (Sykehuset Østfold), Norway Study period: 2002–2005.	Cases: 19 mothers delivering prematurely (<259 days of gestation). Controls: 102 mothers of term neonates. Conditions: vaginal delivery; no reported antibiotic use on or after the day of delivery. Fecal samples were collected on the 4th day postpartum.	16S ribosomal RNA gene (V4 region) sequencing. Bioinformatics analysis: QIIME version 1.7.0.	**The maternal-gut-microbiota profiles (4th postpartum day):** Phylum level: women delivering prematurely had more *Bacillota* (87% vs. 81%), but less *Actinomycetota* (6% vs. 10%). Family level: a lower median abundance of the families *Streptococcaceae* and *Bifidobacteriaceae* (NS when applying correction for multiple testing). Four OTUs had a significantly lower abundance in mothers of preterm deliveries compared to term: OTU1142029 (NCBI BLAST: *B. tsurumiense*), OTU4425214 (NCBI BLAST: *S.vestibularis* or *S. salivarius*), OTU4412546 (*Oscillospiraceae*, *Clostridium cluster IV*), and OTU208539 (*Mogibacteriaceae*, *Clostridium Family XIII Incertae Sedis*). **Diversity:** No difference in the maternal beta diversity of preterm vs. full-term deliveries. One IQR increase in Shannon diversity was associated 38% (95% CI: 1%, 61%) lower odds of having a spontaneous preterm birth.
2	Yang H. (2020) [105] PMID: 32917878	Guangzhou Women and Children’s Medical Center, Guangzhou, China Population-level investigation Study period: January 2017–September 2017.	Cases: 1479 pregnant women of Chinese origin from the 9th week of gestation to antepartum (>36th week). Fecal samples were collected at one time point; no longitudinal data. Controls: 1048 nonpregnant women from the Guangdong Gut Microbiome Project (GGMP) cohort.	16S ribosomal RNA gene (V4 region) sequencing. Bioinformatics analysis: QIIME2; the taxonomy was formed by using the Greengenes v.13.8 database.	**Pregnant women exhibited consistent enterotype patterns with normal adults,** which were driven by the abundance of dominant genera (*Bacteroides*, *Prevotella,* and *Ruminococcus*). This enterotype composition was relatively stable across all gestational stages, with a slight reduction in *Ruminococcus*-type in the last stage of pregnancy. Phylum level across pregnancy: *Bacillota* (70.6%) and *Bacteroidota* (17.8%)—relatively stable “core microbiota”. **Microbial alterations associated with gestational age.** Increased with gestational age: [*Ruminococcus*], *Collinsella*, *Megamonas*, and *unclassified-Erysipelotrichaceae*. Decreased with gestational age: *Ruminococcus*, *Dialister*, and *unclassified-Lachnospiraceae*. The most common taxa in the mid-trimester: *Streptococcus*, *Megasphaera*, *unclassified-Clostridiales*, and *Bacteroides*. Enriched at 21–28 weeks: *Streptococcus* and *Megasphaera*. Enriched at 17–24 weeks: *unclassified-Clostridiales.* Reduced at 21–28 weeks: *Bacteroides*. **Diversity:** no significant differences in alpha and beta diversity during the pregnancy period.
3	Hiltunen H. (2021) [103] PMID: 34349229	Turku University Hospital, Turku, Finland Study period: not clear.	Cases: 55 preterm neonates (<35 gestational weeks) and their 51 mothers. Controls: 25 spontaneously born full-term neonates. Fecal samples were collected during the first 3–4 postpartum days.	16S ribosomal RNA gene (V3–V4 region) sequencing Bioinformatics analysis: QIIME2, the taxonomy was formed by using the Greengenes v.13.8 database	**The maternal-gut-microbiota profiles (within 3–4 postpartum days):** Phylum level: *Bacillota* (62.4%) was the dominant taxa, followed by *Bacteroidota* (29.6%) and *Actinomycetota* (4.3%). Family level: *Bacteroidaceae* (22.5%), *Lachnospiraceae* (22.4%), and *Oscillospiraceae* (21.6%). **Relationship of microbiota with clinical characteristics:** - The mothers who had received intrapartum antibiotics had a higher abundance of *Bacillota*, *Fusobacteriota*, *Pseudomonadota*, and *Actinomycetota.* - The mothers not receiving intrapartum antibiotics had a higher abundance of *Porphyromonadaceae*. - Mothers with vaginal delivery presented a higher abundance of *Roseburia* and mothers without antibiotic treatment a higher abundance of *Macellibacteroides.* **Diversity:** No differences in alpha or beta diversity in relation to gestational age or mode of delivery. Significant differences with regard to intrapartum antibiotic use were seen in alpha diversity Faith PD and evenness, Bray–Curtis, and unweighted UniFrac beta diversity. **Contribution of the maternal to neonatal microbiota** was higher in neonates born spontaneously as compared to those born after iatrogenic preterm delivery. Gestational age or mode of delivery did not affect the extent to which the maternal gut microbiota contributed to the preterm gut microbiota.
4	Li D. (2021) [113] doi:10.1016/j.medmic.2021.100046	Zhujiang Hospital, Southern Medical University, China Study period: July 2020–January 2021	Cases: - Preterm group: 15 preterm neonates (after 28th but before 37th gestational week) and their mothers. - Term group: 11 term neonates (37–42 gestational weeks) and their mothers. Controls: blank, distilled water, and air sample swabs. Samples collected: neonates—oral and rectal (within 24 h of birth); mothers—vaginal and rectal (at admittance for delivery).	16S ribosomal RNA gene (V3–V4 region) sequencing Bioinformatics analysis: QIIME version 1.9.1	**The maternal-gut-microbiota profiles (at admittance for delivery):** Phylum level: no significant differences between mothers of preterm and term neonates—*Bacillota* (38.6% vs. 45.2%, preterm and term, respectively), *Bacteroidota* (44.8% vs. 36.2%, respectively), *Pseudomonadota* (10.5% vs. 12.7%), *Actinomycetota* (1.4% vs. 3.4%, respectively). Genus level: the abundance of *Rothia* and *Gemella* was considerably decreased in mothers delivered prematurely. **Diversity:** No difference in the maternal gut microbiome. The preterm group had higher alpha diversity in the maternal vaginal microbiota than the term group, and there were more species in the maternal vaginal microbiota of the preterm group, as opposed to the maternal gut microbiota. The preterm group had higher beta diversity in vaginal (*p* = 0.003) microbiomes compared to the term group. **Contribution of the maternal to neonatal microbiota:** *Citrobacter freundii*, *Escherichia coli*, *Ralstonia pickettii*, *Prevotella corporis*, *Lactobacillus iners*, *Prevotella disiens*, *Prevotella timonensis*, *Enterococcus faecium*, *Faecalibacterium prausnitzii*, *Corynebacterium amycolatum*, and *Ureaplasma parvum* were detected in neonatal and maternal gut microbiota. Similar species of neonatal oral and maternal vaginal microbiota: *Lactobacillus iners*, *Lactobacillus johnsonii*, *Ralstonia pickettii*, *Neisseria subflava*, *Ureaplasma parvum*, *Rothia mucilaginosa*, *Prevotella timonensis*, *Escherichia coli*, *Streptococcus salivarius subsp. thermophilus*, *Prevotella bivia*, *Prevotella colorans*, and *Enterococcus faecium.*

This table shows the names of genera and species as given by the authors. Phylum, order, and family names were changed to the new nomenclature according to NCBI Taxonomy as required under the International Code of Nomenclature for Prokaryotes (ICNP).

**Table 3:** Features of the intestinal microbiome in mothers with pre-eclampsia (PE).

№	Authors, PMID	Setting and Time Period	Participants and Study Design	Methods	Features of Microbiome
1	Liu J. (2017) [107] PMID: 27988814	Department of Obstetrics of the First Affiliated Hospital of Jinan University, Guangzhou, China. Study period: 2014.	Cases: 26 women newly diagnosed with PE in the third trimester. Control groups: - I: 24 women in the first trimester (11–14 weeks). - II: 24 women in the second trimester (24–28 weeks). - III: 26 women in the third trimester.	16S ribosomal DNA gene (V4 region) sequencing. Bioinformatics analysis: FLASH software was used to assemble the reads into tags, and the USEARCH package was used to cluster tags into OTUs.	**The maternal-gut-microbiota profiles:** Phylum level: women with PE had an increased level of *Cyanobacteriota* (1.07%), which was the fifth most abundant phylum. Healthy women in the third trimester (compared to women in the first and second trimesters) had significantly more *Mycoplasmatota* (0.30%), and *Verrucomicrobiota* almost disappeared. Species level: *Clostridium perfringens* and *Bulleidia moorei* had a significantly higher and *Coprococcus catus* had a significantly lower relative abundance in women with PE compared to healthy women in the third trimester. **Diversity:** There was no statistical significance in alpha diversity among the four groups.
2	Lv L.-J. (2019) [108] PMID: 31297341	Guangdong Women and Children Hospital, Guangzhou, China. Study period: January 2017–December 2017.	Cases: 78 women newly diagnosed with PE with severe effects in their third trimesters. Controls: 72 healthy pregnant women. Fecal-sample collection: in the third trimester and at 1 and 6 weeks postpartum.	16S ribosomal DNA gene (V4 region) sequencing. Bioinformatics analysis: OTU taxonomically classified using Greengenes database v13.8 by QIIME2.	**The maternal-gut-microbiota profiles:** Phylum level: *Fusobacteriota*, *Mycoplasmatota*, and *Verrucomicrobiota* were decreased in PE women at the antenatal time point. Genus (and species) level: eight genera were enriched in antenatal PE samples—*Blautia* (*Blautia* spp., 84.9%) and *Ruminococcus2 (R. gnavus*, 54.7%), followed by *Bilophila (B. wadsworthia*, 100%) and *Fusobacterium (F. nucleatum*, 100%), represented the major variances in PE microbiomes. Genera depleted in antenatal PE samples: *Faecalibacterium*, *Gemmiger*, *Akkermansia*, *Dialister*, and *Methanobrevibacter* (mostly consisted of *F. prausnitzii*, *G. formicilis*, *A. muciniphila*, an unclassified *Dialister spp.*, and *M. smithii*, respectively). **Diversity:** no significant differences in alpha and beta diversity were detected during the antepartum and postpartum periods. **Relationship of microbiota with clinical characteristics:** the systolic and diastolic blood pressure levels were positively correlated with PE-enriched genera (*Anaerococcus*, *Ruminococcus2*, *Fusobacterium*, and *Oribacterium*). The fetal features (e.g., birth weight) were positively correlated with PE-depleted genera. IL-6 was positively associated with *Blautia* and *Bilophila*, and negatively associated with *Faecalibacterium.*
3	Wang J. (2019) [119] PMID: 31850241	Peking University Third Hospital, Beijing, China. Study period: January 2018–December 2018.	Cases: 48 women with PE. Controls: 48 healthy pregnant women. Fecal samples were collected in the third trimester. Analysis of fecal and plasma lipopolysaccharide (LPS) and plasma trimethylamine-N-oxide (TMAO) concentration levels.	16S ribosomal DNA gene (V4 region) sequencing. Bioinformatics analysis: Sequence analysis was performed using Uparse software. The Silva Database based on the Mothur algorithm was used to annotate the taxonomic information. Alpha and beta diversity metrics were calculated using the QIIME v1.7.0.	**The maternal-gut-microbiota profiles (third trimester):** Phylum level: the relative abundance of *Bacillota* was decreased in the PE group (51.6% vs. 59.6%, respectively, *p* < 0.05). The abundance increased in PE compared with controls: *Bacteroidota* (40.5% vs. 34.8%, *p* < 0.05), *Pseudomonadota* (4.5% vs. 2.5%, *p* < 0.05), and *Actinomycetota* (2.9% vs. 1.8%, *p* < 0.05). The abundances were lower in PE: *Bacillota*, *Clostridia*, *Eubacteriales*, *Oscillospiraceae*, *Rikenellaceae, Faecalibacterium*, *Alistipes*, and *Bacteroides_stercoris.* The abundances were higher in PE: *Bacteroidota*, *Pseudomonadota*, *Actinomycetota*, *Bacteroidia*, *Gammaproteobacteria*, *Enterobacterales*, *Enterobacteriaceae*, *Bacteroides_coprocola*, and *Bacteroides_fragilis*. **Diversity:** Alpha diversity was lower in the PE group (but NS). Beta diversity (by UniFrac distance) was different (ANOSIM analysis, *p* = 0.011). **LPS and TMAO levels.** The fecal and plasma LPS concentrations and plasma TMAO concentrations were higher in PE women.
4	Chen X. (2020) [118] PMID: 31900289	Department of Obstetrics of the Nanfang Hospital, Southern Medical University, Guangzhou, China. Study period: March 2017–March 2018.	Cases: 67 women with PE (40 of them with severe PE)—21 with an early onset of PE and 46 with a late onset of PE. Controls: 85 normotensive pregnant women. Fecal samples were collected in the third trimester. Experimental part of the study: fecal-microbiota transplantation in an antibiotic-treated mouse model.	16S ribosomal RNA gene sequencing. Taxonomic groups were based on the Greengenes Database v.13.8 using QIIME v.1.9.1.	**The maternal-gut-microbiota profiles (third trimester):** Genus level: *Clostridium*, *Dialister*, *Veillonella*, and *Fusobacterium* were significantly increased, whereas *Lachnospira*, *Akkermansia*, and *Faecalibacterium* were depleted in PE, but there were no differences between severe and not-severe PE. **Diversity:** Alpha diversity was markedly decreased in the PE group. The microbiome of the PE group differed significantly from the normal pregnancy group (using the unweighted UniFrac distance). There were no significant differences between the PE with and without severe features or between the early and late onset of PE subgroups for both alpha diversity and beta diversity. **Relationship of microbiota with clinical characteristics:** *Veillonella* and *Fusobacterium* were correlated for the systolic and diastolic blood pressure, proteinuria, oedema levels, alanine aminotransferase, aspartate aminotransferase, serum creatinine, and albumin. The correlations for *Lachnospira*, *Akkermansia*, and *Faecalibacterium* were opposite. No bacteria were correlated with clinical parameters when testing only on the PE group. **PE patients’ fecal microbiota transplantation of induced PE phenotype in mice:** At 6 weeks after transplantation, mice had higher pregestational systolic blood pressure, which was further elevated. PE-transplanted mice had increased proteinuria, embryonic resorption, and lower fetal and placental weights. Their T regulatory/helper-17 balance in the small intestine and spleen was disturbed with more severe intestinal leakage.
5	Wang J. (2020) [114] PMID: 32265423	Nanjing Maternity and Child Health Care Hospital, Nanjing, China. Study period: January 2018–December 2018.	Cases: 25 women who subsequently developed PE. Controls: 25 healthy pregnant women. Fecal samples were collected in the second trimester (20–24 weeks) and third trimester (32–34 weeks).	16S ribosomal RNA gene (V4 region) sequencing. Bioinformatics analysis: sequence analysis was performed using Uparse software. The Silva Database based on the Mothur algorithm was used to annotate the taxonomic information. Alpha and beta diversity metrics were calculated using QIIME v1.9.1.	**The maternal-gut-microbiota profiles:** Phylum level in dynamics (second to third trimester): PE patients had a lower relative abundance of *Bacteroidota* in second trimester than that of the third trimester. The relative abundances of *Pseudomonadota* and *Mycoplasmatota* significantly decreased in controls from the second to third trimester. Family level: *Enterobacteriaceae* decreased in controls. Phylum level in each trimester: second trimester—the relative abundances of *Bacillota*, *Bacteroidota*, *Actinomycetota*, *Pseudomonadota*, and *Mycoplasmatota* showed no significant differences between the PE and the control group. Third trimester—the abundance of *Bacillota* was significantly lower in the PE group than in controls (mean 60.6% vs. 75.5%, respectively, *p* < 0.05). *Bacteroidota* and *Pseudomonadota* were higher in the PE group than in controls (median 31.09% vs. 18.24%, respectively, *p* < 0.05; 1.52% vs. 0.64%, respectively, *p* < 0.05). There were no significant differences in the abundances of *Actinomycetota*, and *Mycoplasmatota* between the two groups. LefSe: the relative abundances of the phylum *Bacteroidota*, class *Bacteroidia,* and order *Bacteroidales* were increased in the PE group, *Bacillota*, class *Clostridia*, order *Eubacteriales*, and genus unidentified *Lachnospiraceae* were decreased in the PE group in the third trimester. Family level at the third trimester: the abundance of *Enterobacteriaceae* was significantly higher in the PE group than in the control group (median, 0.75% vs. 0.01%, respectively, *p* < 0.05). **Diversity:** the Shannon and Simpson indices in the PE group were slightly lower than those in the control group in the second and third trimesters, but NS.
6	Miao T. (2021) [117] PMID: 34262418	Department of Obstetrics and Gynecology in Changzhou Maternity and Child Health Care Hospital (which is affiliated to Nanjing Medical University), Guangzhou, China. Study period: October 2017–April 2018.	Groups (periods of fecal-sample collection): Cases: 12 women with PE (35.2 ± 2.0 weeks). Controls: eight women without PE (34.8 ± 4.6 weeks). Fecal samples were collected in the third trimester.	16S ribosomal RNA gene (V4 region) sequencing. Bioinformatics analysis: OTU taxonomically classified using Greengenes database v201305 by QIIME v1.8.0.	**The maternal-gut-microbiota profiles (third trimester):** Phylum level: women with PE had decreased abundance of *Actinomycetota* compared to the control group (*p* = 0.042). The control group had an increased abundance of *Mycoplasmatota*. Family level: The relative abundance of *Bifidobacteriaceae* was lower in the PE group (3.75%) than in the control group (12.76%) (*p* = 0.039). The relative abundance of *Oscillospiraceae*, *Prevotellaceae*, and *Coriobacteriaceae* in the PE group was decreased compared to the control group (NS). Genus level: *Blautia* was increased significantly in the PE group in comparison with that in the control group (19.13% vs. 9.71%, respectively, *p* = 0.026). *Ruminococcus* was increased in the PE group compared to the control group (10.32% vs. 6.11%, respectively, *p* = 0.048). There was a reduction in *Bifidobacterium* in the PE group compared to the control group (*p* = 0.038). Downward trend in *Faecalibacterium*, *Roseburia,* and *Prevotella* in the PE group compared with that in the control group (NS). **Diversity:** There was no statistical significance in the alpha diversity among groups or the differences in beta diversity between groups. **Relationship of microbiota with clinical characteristics:** The relative abundance of *Blautia* was positively correlated with maternal age, pregestational weight, hematocrit, levels of C-reactive protein, triglyceride (*p* < 0.05 for all), and low-density lipoprotein cholesterol (*p* < 0.01). The relative abundance of *Ruminococcus* was positively correlated with the pregestational weight, pregestational BMI, antepartum weight, antepartum BMI, lipopolysaccharide-binding protein, and triglyceride (*p* < 0.05 for all). The relative abundance of *Bifidobacterium* was significantly negatively correlated with the systolic and diastolic blood pressure, levels of cholesterol and aspartate aminotransferase (*p* < 0.05 for all), and triglyceride level (*p* < 0.01).
7	Huang L. (2021) [121] PMID: 34607559	Changsha Hospital for Maternal and Child Health Care, Hunan, China. Study period: not clear.	Cases: - 26 pregnant women with PE. - 25 pregnant women with abnormal placental growth. Controls: - 28 healthy pregnant women. - 21 healthy women. Fecal samples were collected in the third trimester.	16S ribosomal RNA gene (V4 region) sequencing. Bioinformatics analysis: qualified paired-end reads were matched, dereplicated, clustered, and chimera-filtered using VSEARCH (v2.4.4) against the SILVA138 database and then OTUs were assembled using QIIME2.	**The maternal-gut-microbiota profiles (the third trimester):** Phylum level: the percentage of TM7 (candidatus *Saccharibacteria*) was significantly increased in the abnormal-placental-growth group. Genus level: The relative abundances of *Prevotella*, *g_WAL_1855D*, *g_1_68*, *Porphyromonas*, *Varibaculum*, and *Lactobacillaceae* were significantly decreased in the PE group compared with the normal-pregnancy group. *Prevotella*, *g_1_68*, *Porphyromonas*, *Lactobacillaceae*, *Mobiluncus*, *Campylobacter*, and *Peptostreptococcus* were decreased in the abnormal-placental-growth group compared with the normal-pregnancy group. The ratio of number of subject *Lactobacillaceae*/all and the relative abundance of *Lactobacillaceae* were significantly higher in the normal-pregnancy group. **Diversity:** Alpha diversity exhibited no statistical differences. There was a significant difference in beta diversity (Bray–Curtis distance and Adonis) in the bacterial composition only in the abnormal-placental-growth group compared with the nonpregnant group (*p* = 0.043). The gut microbiota compositions of the abnormal-placental-growth group, the PE group, or both groups of abnormal pregnancies were significantly shifted compared with that of the normal pregnancy group (Adonis, *p* = 0.002, *p* = 0.015, *p* = 0.001, respectively). The PE group was significantly different from the normal-pregnancy group. The abnormal-placental-growth group had the highest number of unique OTUs. **Relationship of microbiota with clinical characteristics:** *Lactobacillaceae* (OTU 255) was significantly negatively related to diastolic blood pressure in the PE and normal-pregnancy groups.
8	Jin J. (2022) [123] PMID: 35950704	Liaocheng, China Study period: 2017–2022. Samples collected:	Cases: 92 women with PE (37.69 ± 2.4 weeks). Controls: 86 healthy pregnant women (37.05 ± 3.2 weeks). Fecal samples were collected in the third trimester. Analysis of SCFA in feces, serum, and placentas. Experimental part of the study: fecal-microbiota transplantation in mice.	16S ribosomal RNA gene (V3–V4 regions) sequencing. Bioinformatics analysis: The dereplicated sequence reads were denoised into ASVs. The phylogenetic affiliation was analyzed using the USEARCH 10 SINTAX algorithm against the RDP training-set database.	**The maternal-gut-microbiota profiles:** Genus and species level: the abundances of many intestinal SCFA-producing bacteria were significantly reduced in PE (*Alistripes*, *Fusicatenibacter*, *Coprobacter*, *Oscillibacter*, *Clostridium_XIVb*, and *Clostridium_XV*). PE and controls could be distinguished only based on the abundances of *Akkermansia* and *Oscillibacter* with 89.7% accuracy. **Diversity:** the alpha and beta diversity and *Bacillota*/*Bacteroidota* ratio of the gut microbiota in PEs were significantly changed, suggesting gut dysbiosis. **Relationship of microbiota with clinical characteristics:** *Akkermansia* was reduced in PE and negatively correlated with blood pressure and urine protein. *A. muciniphila* (SCFA-producing bacteria) was significantly reduced in the feces of PE women, and was significantly positively correlated with the levels of propionate and butyrate in the placenta. *Akkermansia* abundance was positively correlated with the levels of fecal 2-Arachidonoylglycerol and serum IL-10 but negatively correlated with the serum levels of lipopolysaccharide and IL-17. **Fecal, placental, and serum levels of propionic and butyric acids were significantly reduced in PE** and positively correlated with each other. Placental levels of propionic and butyric acids negatively correlated with blood pressure and urine protein levels. The abundances of bacteria were very low in the placentas, did not differ and are likely to have originated from contamination during the experimental procedure. The peripheral blood Treg/Th17 ratio was significantly decreased in PE. The abnormalities of peripheral Treg and Th17 cells in PEs were closely related to the intestine. **The intestinal-barrier damage in PE was revealed.** The serum level of lipopolysaccharide was considerably increased in PE. The fecal level of 2-Arachidonoylglycerol, a protector of the intestinal barrier, was decreased. **PE patients’ fecal microbiota transplantation of induced pre-eclamptic phenotype in rats.** *Akkermansia muciniphila*, propionate, or butyrate significantly alleviated the symptoms of pre-eclamptic rats. Propionate promoted trophoblast invasion, thereby improving spiral arterial remodeling.
9	Li P. (2022) [124] PMID: 36380372	A two-sample Mendelian randomization study.	Analysis of gut microbiota from GWAS meta-analysis conducted by the MiBioGen consortium (n = 18,340 individuals) and the summary statistics of PE from the FinnGen consortium R7 release data (5731 cases and 160,670 controls).	16S ribosomal RNA gene sequencing targeting variable regions V4, V3–V4, and V1–V2 (MiBioGen consortium).	**Maternal microbial profiles:** Genus level:*Collinsella* (OR 0.77), *Enterorhabdus* (OR 0.76), *Eubacterium (ventriosum group)* (OR 0.76), *Lachnospiraceae (NK4A136 group)* (OR 0.77), and *Tyzzerella 3* (OR 0.85) were found to be associated with PE. *Bifidobacterium* had a protective effect against PE.
10	Lv L.-J. (2022) [125] PMID: 36189343	Guangdong Women and Children Hospital, Guangzhou, China. Study period: not clear.	Cases: 40 women with severe PE (246 ± 25 gestational days). Controls: 37 healthy pregnant women (273 ± 11 gestational days). Fecal samples were collected in the third trimester.	Shotgun metagenomic sequencing. Taxonomic classification of the bins was realized based on the GTDB-Tk toolkit (v1.4.0) by assigning the sequences of each bin to the Genome Taxonomy Database (v. r95). The taxonomic name of the bins was manually modified to accord with traditional nomenclatures following the National Center for Biotechnology Information (NCBI) taxonomy.	**The maternal-gut-microbiota profiles (the third trimester):** Family level: *Lachnospiraceae* and *Coriobacteriaceae* were significantly enriched in PE patients compared with healthy controls; *Bacteroidaceae* were markedly depleted in the PE patients. Genus and species levels: - PE-enriched species: *Blautia* (unknown at the species level), five members of *Pauljensenia* (*P. bouchesdurhonensis* and four uncultivated species), five members of *Ruminococcus* (containing *R. gnavus* and four uncultivated species), and *Fusobacterium ulcerans.* - Control-enriched species: 14 members of *Bacteroidaceae*, containing 7 *Bacteroides* spp., 4 *Phocaeicola* spp., *Prevotella bivia*, *Paraprevotella clara*, *Barnesiella intestinihominis*, and also *Akkermansia muciniphila* and *Bilophila wadsworthia*. PE-enriched species (*Olsenella* sp. M220, *Ruminococcus* sp. M094, *Blautia* sp. M090, and *Senegalimassilia anaerobia* M253), as well as several control-enriched species (*Flavonifractor plautii* M314, *Bacteroides uniformis* M403, and *Bacteroides* sp. M398), featured the highest score for the discrimination of PE patients and healthy controls (AUC = 0.805). **Diversity:** Alpha diversity exhibited no statistical differences. Beta diversity (Bray–Curtis distance) revealed an alteration of the gut microbial structure between PE patients and the control group. The PE status explained 1.4% of the microbial variations (permutated *p* = 0.005).
11	Lin H. (2022) [126] PMID: 36364844	Hunan Provincial Maternal and Child Health Hospital, Changsha, China. Study period: March 2017–March 2018.	Cases: 35 women with pregnancy-induced hypertension (PIH) (15 of them with PE) (13.15 ± 3.26 weeks, initially). Controls: 35 healthy pregnant women (12.70 ± 0.86 weeks, initially). Fecal samples were collected in the first, second, and third trimesters, at delivery and during postpartum period.	Metagenomic sequencing. Taxonomic annotation and functional annotation realized by MetaPhlAn (v. 2.1.0) and HUMAnN (v. 2-0.11.0).	**The maternal-gut-microbiota profiles:** Phylum level: PIH patients had a higher abundance of *Bacillota* and a lower abundance of *Bacteroidota* and *Verrucomicrobiota.* Species level: - Higher abundance in PIH: *Eubacterium rectale* and *Ruminococcus bromii*. - Lower abundance in PIH: *Alistipes putredinis*, *Bacteroides vulgatus*, *Ruminococcus torques*, *Oscillibacter unclassified*, *Akkermansia muciniphila*, *Clostridium citroniae*, *Parasutterella excrementihominis*, and *Burkholderiales bacterium_1_1_47.* **Diversity:** Alpha diversity exhibited no statistical differences. Beta diversity between the PIH and control groups differed statistically. **Relationship of microbiota with clinical characteristics:** The abundance of *A. putredinis* was negatively correlated with early uric acid and early systolic and diastolic blood pressure, and was positively correlated with low-density lipoprotein cholesterol. The abundance of *B. vulgatus* was negatively correlated with early BMI, waist, early ALT, early uric acid, early weight, early AST, insulin, γ-glutamyltransferase (GGT), and early systolic and diastolic blood pressure. The abundance of *O. unclassified* and *A. muciniphila* was negatively correlated with early systolic and diastolic blood pressure, and *A. muciniphila* was positively correlated with GGT. The abundance of *C. citroniae* was negatively correlated with early diastolic blood pressure.

This table shows the names of genera and species as given by the authors. The phylum, order, and family names were changed to the new nomenclature according to NCBI Taxonomy as required under the International Code of Nomenclature for Prokaryotes (ICNP). To date, some taxa have been reclassified or renamed. The correct names of these taxa are given in parentheses: *Bulleidia moorei* (*Solobacterium moorei*).

**Table 4:** Features of the intestinal microbiome in preterm neonates.

№	Authors, PMID	Setting and Time Period	Participants and Study Design	Methods	Features of Microbiome
1	Zhou Y. (2015) [161] PMID: 25741698	Brigham and Women’s Hospital, Boston, MA, USA. Study period: not clear. NEC stage: II–III.	Cases: 12 neonates with NEC (GA of 27.8 (24–31) weeks). Age of NEC: early (≤DOL 22) vs. late (>DOL 22). Controls: 26 neonates without NEC (GA of 27.9 (24–31) weeks). Fecal samples were collected with a median sampling interval of 3 days.	16S ribosomal RNA gene (V3–V5 regions) sequencing. Taxonomic groups were based on the Ribosomal Database Project (RDP) naive Bayesian classifier (version 2.5, training set 9)	**Gut microbiota profiles:** **Early-onset NEC (≤DOL 22):***Clostridium sensu stricto* (*Clostridia*) were significantly higher in proximity to NEC onset. **Late-onset NEC (>DOL 22):** *Escherichia/Shigella* (*Gammaproteobacteria*) was significantly higher in cases than controls six days before NEC onset. *Cronobacter* (*Gammaproteobacteria*) was significantly higher 1–3 days prior to NEC onset. **Diversity:** The richness and Shannon diversity from the samples before NEC onset increased significantly over the 2 months of life. Neonates without NEC had a higher richness (but not Shannon diversity). NEC samples tended to have lower microbial diversity than controls, and they also had marginally more antibiotic usage than the controls.
2	Heida F.H. (2016) [134] PMID: 26787171	University Medical Center of Groningen, Groningen, The Netherlands. Study period: October 2013–February 2014. NEC stage: II–III.	Cases: 11 neonates with NEC (GA of 27 (24–29) weeks). Age of NEC: 12.5 (4–43). Controls: 22 neonates without NEC (GA of 26 (24–29) weeks). Fecal-sample collection: at 1 (0–4) day of life (meconium); then, the two samples were collected in the week prior to the onset of NEC (when available).	16S ribosomal RNA gene (V3–V4 regions) sequencing. Taxonomic identification down to the family and genus levels with QIIME. Sequence identification to the species level with ARB (SILVA rRNA database).	**Gut microbiota profiles:** **NEC group:** There was a higher abundance of *Clostridium perfringens* and *Bacteroides dorei*, and a lower abundance of *Clostridioides difficile* in the meconium. Prior to disease onset, NEC cases remained dominated by *Enterobacteriaceae* or developed an aberrant microbiota composition that formed a distinct cluster, consisting of *C. perfringens* and *B. dorei*. **Controls:** There was a shift from a more *Enterobacteriaeceae*-dominated microbiota into one with more staphylococci (19%) and other lactate-producing bacilli. **Diversity:** This was not associated with NEC development. **Maternal factors:** The amount of breast milk as a percentage of the total amount of feeding was correlated with an increase in lactate-producing bacilli and a decrease in Gram-negative species, which include the *Enterobacteriaceae* and *Bacteroidaceae*.
3	Warner B.B. (2016) [152] PMID: 26969089	- St. Louis Children’s Hospital; - Children’s Hospital at Oklahoma University Medical Center; - Kosair Children’s Hospital, USA. Study period: July 2009–September 2013. NEC stage: II–III.	Cases:28 neonates with NEC (GA of 26.0 (24.7–27.9) weeks) Age of NEC: 24 (19–48). Controls: 94 Neonates without NEC (GA of 27.0 (25.9–28.7) weeks) Fecal-sample collection: all stools of neonates were collected up to and including the day before NEC was diagnosed or 60 days of age.	16S ribosomal RNA gene (V3–V5 regions) sequencing Read classification using the Ribosomal Database Project naive Bayesian classifier version 2.5, training set 9.	**Gut microbiota profiles:** **NEC group:** A significant increase in *Gammaproteobacteria* over time, and a decrease in *Negativicutes* and the combined *Clostridia–Negativicutes* class (in infants with a GA < 27 weeks). **Controls:** Associated with an abundance of *Negativicutes*, and the combined *Clostridia–Negativicutes* class (in infants with a GA < 27 weeks). The *Clostridia–Negativicutes* class increased over time (in infants with a GA > 27 weeks). **Diversity:** The alpha diversity significantly increased in the stools of controls, but not in NEC patients (the diversity decreased over time). **Associations with clinical features:** A greater GA at birth was associated with higher proportions of *Negativicutes* and the combined *Clostridia–Negativicutes* class, and lower proportions of *Bacilli*. Vaginal birth was associated with lower proportions of *Bacilli*. Greater antibiotic exposure was associated with higher proportions of *Bacilli*, and lower proportions of *Clostridia*, and the combined *Clostridia–Negativicutes*.
4	Ward B.V. (2016) [154] PMID: 26997279	Two level III neonatal intensive care units (NICUs), Cincinnati, OH, USA. One level III NICU in Birmingham, AL, USA. Study period: December 2009–July 2012. NEC stage: II–III.	Cases: 27 neonates with NEC (GA of 26 (23–28) weeks). Age of NEC: 26 (10–39). Preterm controls: 117 neonates without NEC (GA of 26 (23–29) weeks). Term controls: 22 neonates without NEC (GA 39 (38–41) weeks. Fecal-sample collection in three collection periods (days 3–9, 10–16, and 17–22).	Shotgun metagenomic sequencing. Relative taxonomic abundances were determined with MetaPhlAn version 2.0. Pangenome-based strain-level profiling of *E. coli* from metagenomes, and multilocus sequence type (MLST) analysis.	**Preterm gut microbiome analysis (overall):** Days 3–9: Median relative abundance (MRL): *E. coli* (0.92), *Serratia marcescens* (0.24), *Klebsiella* spp. (0.14), *Streptococcus* sp. GMD4S (0.12), and *E. faecalis* (0.11). Days 10–16: *E. coli* (0.93), *S. marcescens* (0.39), *Veillonella parvula* (0.33), *Klebsiella* spp. (0.19), *K. oxytoca* (0.11), and *Streptococcus* sp. GMD4S (0.12). Days 17–22: *E. coli* (0.81), *Klebsiella* spp. (0.48), *V. atypica* (0.21), *E. cloacae* (0.14), *Citrobacter freundii* (0.13), and *Streptococcus* sp. GMD4S (0.12). Infants with high antibiotic treatment were specifically enriched in *E. coli* relative to low-treatment infants who were enriched in the order *Eubacteriales*, genus *Veillonella*, and *Klebsiella* spp. Infants who developed NEC had less *Veillonella* and were specifically enriched in *E. coli*. **NEC group:** Colonization by uropathogenic *E. coli* (UPEC) was a highly significant risk factor for the development of NEC. UPEC was even more strongly correlated with mortality as an outcome. NEC cases with *Klebsiella* dominance were also observed. **Diversity** was lower among infants receiving a high level of antibiotic administration. Alpha diversity was higher at days 17–22 in preterm controls but decreased in infants who developed NEC.
5	Dobbler P.T. (2017) [148] PMID: 29187842	Neonatology Section of Hospital de Clínicas de Porto Alegre, Brazil. Study period: not clear. NEC stage: not clear.	Preterm neonates with a GA ≤ 32 weeks. Cases: 11 neonates with NEC Age of NEC: 8.0 (5.0–13.0). Controls: 29 neonates without NEC. Fecal-sample collection: with the first stool (meconium) weekly up to the 5th week of life.	16S ribosomal RNA gene (V4 region) sequencing. Taxonomic classification was carried out in QIIME based on the UCLUST method against the Greengenes 13.5 database.	**NEC group:** There was an indeterminate pattern of microbial succession (called here as ‘chaotic’ or ‘abnormal’ pattern). There was a higher abundance of *Pseudomonadota* and lower abundance of *Bacillota* during week 3. There was an overall higher average abundance of *Actinomycetota* than in controls during weeks 1 and 2. Days 0–4: dominance of *Pseudomonadota* (44.66%) and lower abundance of *Bacteroidota* (35.23%), *Bacillota* (15%), and *Actinomycetota* (1.7%). Days 5–7: an abrupt decrease in *Pseudomonadota* in the NEC group. Weeks 2–3: a bloom of *Pseudomonadota;* from then, a steady decline through weeks 4 and 5 that coincided with an increase in *Bacillota*. The early detection of a high dominance of *Enterobacteriaceae*, especially *Citrobacter koseri* and *Klebsiella pneumoniae*, a lack of *Lactobacillaceae*, low diversity, and altered microbial–microbial associations during the first days of life could be indicators of NEC risk in preterm infants. **Controls:** a steady increase in *Bacillota* overlapping with a decline in *Pseudomonadota*, *Bacteroidota*, and *Actinomycetota* until week 4 followed by a sudden re-emergence of *Pseudomonadota*. Days 0–4: a high abundance of *Pseudomonadota* (40.07%) and *Bacteroidota* (36.35%), and a low abundance of *Bacillota* (13.14%) and *Actinomycetota* (2.47%). Days 5–7: a dominance of *Bacillota* (52.64%) and *Pseudomonadota* (31.43%) with a low abundance of *Bacteroidota* (13.47%) and *Actinomycetota* (0.54%). Weeks 2–3: a steady increase in *Bacillota* overlapping with a decline in *Pseudomonadota*, *Bacteroidota*, and *Actinomycetota* until week 4 followed by a sudden re-emergence of *Pseudomonadota*. A community of obligate anaerobes was highly influential in the intestine of controls during the first four days of life and appeared to control the proliferation of *Enterobacteriaceae*. **Diversity:** patients with NEC tended to have low alpha diversity and high dominance compared to the controls (significant during the 3rd week). Microbial dominance at the 3rd week was higher in the NEC cases than in the controls.
6	Rozé J.-C. (2017) [157] PMID: 28659297	EPIFLORE Study, France Study period: 2011. NEC stage: II–III.	Cases: 14 neonates with NEC (GA of 28.4 ± 1.9 weeks). Age of samples collected at onset: 25.8 ± 16.7. Controls: 73 neonates without NEC (GA of 28.5 ± 1.7 weeks). Fecal-sample collection at DOL 7, 28, and at NEC onset (3 (0–7) days after onset)/at discharge in controls.	16S ribosomal RNA gene (V3–V4 regions) sequencing. Culture methods.	**NEC group (culture methods):** The *Clostridium* genus was significantly associated with NEC: (86% vs. 35%, OR: 11.3). At the species level, *C. neonatale* (50% vs. 11%, OR 5.5) and *Staphylococcus aureus* (57% vs. 13%, OR 7.1) were significantly associated with NEC. **NEC group (16S rRNA sequencing)**: Colonization with bacteria from the *Clostridium sensu stricto* genus tended to be associated with NEC (20.6% vs. 11.7%, *p* = 0.08) and with a higher proportion of *C. neonatale,* together with *C. butyricum*. *Gammaproteobacteria* were also differentially represented with a trend toward lower proportions of *Klebsiella* and *Citrobacter* and an association with some specific bacterial operational taxonomic units related to either clostridia or *Gammaproteobacteria* in NEC infants.
7	Wandro S. (2018) [136] PMID: 29875143	Children’s Hospital, Orange County, Orange, CA, USA. Study period: 2011–2014. NEC stage: not clear.	32 preterm neonates with birth weights of 620–1570 g. Cases: three neonates with NEC; eight neonates with late-onset sepsis. Age of NEC: 27, 31, and 41. Controls: 21 healthy neonates. Fecal-sample collection: between days 7 and 75 of life. The sampling times and numbers of fecal samples varied. Gas chromatography–mass spectrometry was used.	16S ribosomal RNA gene (V3–V4 regions) sequencing Taxonomy was assigned using QIIME and the Greengenes 13_8 database.	**There were no clear signatures of microbiome composition linked to NEC or sepsis.** Longitudinal samples from individual infants remained highly personalized over several weeks. Preterm infant microbiomes were shaped by shared exposures, especially to antibiotics, leading to the dominance of antibiotic-resistant facultative anaerobes *(Enterococcus* spp.). The anaerobic, milk-degrading bifidobacteria were largely absent, even in preterm infants with access to breast milk. **Diversity:** The difference in alpha diversity was associated with antibiotic use. Only vaginally born infants were colonized by *Bacteroides* (4 out of 9 infants), while none of the 22 infants born via C-section were colonized. **Metabolite profiles:** the metabolite profiles varied over time (individually) and were not associated with NEC or late-onset sepsis.
8	Romano-Keeler J. (2018) [156] PMID: 30365522	Monroe Carell Jr. Children’s Hospital, Vanderbilt, TN, USA. Study period: not clear. NEC stage: surgical NEC.	Cases: 12 surgical patients with NEC (GA of 25–33 weeks). Controls: 14 surgical patients without NEC (GA of 24–39 weeks). Age at surgery: 5–46. Sample collection: intestinal tissue during surgery, patient’s first post-operative stool, or by scraping surgical tissue.	16S ribosomal RNA gene (V1–V3 regions) sequencing. Sequences were aligned to the SILVA database release 123 and taxonomically classified with the Ribosomal Database Project (RDP) classifier 11.	**NEC group:** Tissue samples—higher abundances of *Staphylococcus* and *Clostridium sensu stricto I*. Fecal samples—a higher abundance of *Staphylococcus* and lower abundances of *Actinomyces* and *Corynebacterium*. **Diversity:** The fecal microbial richness and diversity tended to be lower in NEC patients (*p* = 0.078). The tissue microbial richness was lower (*p* < 0.05); the alpha diversity tended to be lower than in controls (*p* = 0.081). There was distinct beta diversity in the tissue samples of NEC patients vs. controls. **Metabolic pathways** in NEC patients were related to signatures of infectious diseases (bacterial toxins) and Staphylococcus aureus infection was enriched in NEC tissue samples compared to non-NEC tissues.
9	Olm M.R. (2019) [151] PMID: 31844663	University of Pittsburgh Medical Center Magee-Womens Hospital, Pittsburgh, PA, USA. Study period: 5-year period. NEC stage: not clear. Pre-NEC samples were analyzed.	Cases: 34 neonates with NEC (GA of 28 ± 5.5 weeks). Age of NEC: DOL 9 ± 9.8. Controls: 126 neonates without NEC (GA of 29 ± 2.2 weeks). Fecal-sample collection: longitudinal (average 7.2 samples per patient) until NEC onset within 2 days before NEC diagnosis— “pre-NEC” samples.	Shotgun metagenomic sequencing. To determine the taxonomy of bins, the amino acid sequences of all predicted genes were searched against the UniProt database using the USEARCH ublast command. tRep was used to convert the list of identified taxIDs into taxonomic levels.	The gut microbiomes of all infants were dominated by *Pseudomonadota*, regardless of NEC development. The premature infants had increased *Enterobacteriaceae* and notably low abundances of *Actinomycetota* and *Bacteroidota*. **NEC group:** A low abundance of *Bacillota* and a higher abundance of *Enterobacteriaceae*. However, in pre-NEC samples, the gut microbiomes were not significantly enriched in *Enterobacteriaceae* (the association of *Enterobacteriaceae* and NEC infants may be after the administration of antibiotics to treat NEC). The *K. pneumoniae* strain 242_2 was the most associated with NEC. iRep (bacterial replication rates) values of bacteria were significantly higher in pre-NEC versus control samples. Bacterial replication was stable 4 or more days before NEC, increased daily in the 3 days before diagnosis, and crashed following diagnosis (probably due to subsequent antibiotic administration). The genomes of *Enterobacteriaceae* displayed higher pre-NEC iRep values than bacteria overall. **Diversity:** not clear **Metabolic pathways:** Four factors were significantly associated with pre-NEC samples (taken within 2 days before NEC diagnosis): iRep values overall, genomes encoding specific types of secondary metabolite gene clusters (sactipeptides, bacteriocins, and butyrolactones), *Klebsiella*, and fimbriae cluster 49.
10	Lindberg T. (2020) [150] PMID: 29909714	IV neonatal intensive care unit (NICU), Hartford, CT, USA. Study period: September 2013–September 2015. NEC stage: II–III.	Preterm neonates with a GA of 25.2 (23–27) weeks. Cases: five neonates with NEC. Age of NEC: not clear. Controls: five neonates without NEC. Fecal-sample collection was on a weekly basis beginning with the first bowel movement until patient discharge.	16S ribosomal RNA gene (V4 region) sequencing. Indicator value analysis using the Indicspecies Package was performed to identify microbial species.	**NEC group:** A dominance of *Pseudomonadota* (65.5%), followed by *Bacillota* (28.1%), *Actinomycetota* (5.8%), and other bacteria (0.5%). *Enterobacteriaceae* and *Trabulsiella* were more abundant in NEC patients. **Controls:** There was a dominance of *Bacillota* (55.9%), followed by *Pseudomonadota* (40.8%), *Actinomycetota* (2.9%), and other bacteria (0.5%). *Veillonella* and *Enterococcus* were more abundant in controls. There was a significant reduction in *Pseudomonadota* in early samples compared to those collected at later time points. **Diversity:** Alpha diversity was associated with the day of life (↑ by 0.02 with each day) and antibiotics use (↓ of 0.01 for each additional day).
11	Brehin C. (2020) [171] PMID: 32709038	Purpan Hospital in Toulouse, France. Study period: not clear. NEC stage: I (suspected NEC).	Cases: 11 neonates with NEC (GA of 28.4 (26–31) weeks). Age of NEC I: 12 (4–60) Controls: 21 neonates without NEC (GA 30 (26.4–32) weeks). Fecal samples were collected at the time points of 1–10 days; 11–20 d; 21–30 d; and >30 d. Fecal metabolome analysis.	16S ribosomal RNA gene (V3–V4 regions) sequencing Diseases and host genetic variation linked to NEC-1 associated gut microbiota were identified via MicrobiomeAnalyst.	**Gut microbiota profiles in NEC stage I:** Days 0–10: had a divergent and more homogenous gut microbiota and lower alpha diversity (Chao1). Days 11–20: had a higher abundance of *Streptococcus* species and bacteria from the *Micrococcales* order, lower levels of serine in the fecal metabolome and a higher Chao1 index. Days 21–30: had increased *Staphylococcus* and *Streptococcus* species, high intragroup variance, no difference in the Chao1 index, and no difference in the fecal metabolome. The NEC-1 gut microbiota profile was associated with multiple diseases and was found to be significantly increased in ulcerative colitis and host genetic variation and significantly related to ANP32E, a gene involved in ulcerative colitis. Over 30 days: had an increase in *Raoultella* species. There was no change in the overall microbial diversity indices, but significantly lower levels of ethanol and leucine in the fecal metabolome.
12	Fu X. (2021) [162] PMID: 34012949	First Hospital of Jilin University, Changchun, China. Study period: February 2018–April 2019. NEC stage: II–III.	Cases: 15 preterm neonates with NEC (GA 30.2 ± 1.2 weeks). Controls: 15 preterm neonates without NEC (GA 30.1 ± 1.9 weeks). Fecal samples were collected within 48 h after birth, once per week until the NEC diagnosis, 1–2 weeks after treatment, or 28 days after birth.	16S ribosomal RNA gene (V3–V4 regions) sequencing. Taxonomic groups were based on the Greengenes Database using QIIME2.	**Gut microbiota profiles:** **NEC group:** There was a higher abundance of *Bacteroidota,* and *Actinomycetota* at birth was much higher than that in controls (which continued until NEC occurred). Higher abundance of *Alphaproteobacteria*, *Betaproteobacteria*, *Sphingomonas*, *Lactobacillaceae* at NEC onset. In children who subsequently developed NEC, environmental bacteria were detected in the first 48 h of life: *Dysgonomonas* (lives on surfaces), *Hyphomicrobiales*, *Ralstonia,* and *Pelomonas* (in water sources and hospital ventilators). **Controls:** had a higher abundance of *Gammaproteobacteria*, *Enterobacteriaceae*, and *Clostridiaceae*. **Diversity:** at birth—the Chao1 index of the NEC group was higher, but the Shannon index, Good’s coverage, and Pielou’s evenness index were NS; at NEC onset—the Chao1 index, Shannon index, and Pielou’s evenness index of the NEC group were higher. **Metabolic pathways:** in the NEC group at birth, the pathways involved in the degradation of L-tryptophan and aromatic compounds were upregulated (the species composition was mainly *Ralstonia*).
13	Tarracchini C. (2021) [130] PMID: 34704805	Meta-analysis of metagenomics data from repositories. Data from Croix Rousse University Hospital, Lyon, France were included additionally. Study period: September 2014–November 2014. NEC stage: II–III.	Data from repositories: GA of 23–39 weeks. Cases: 67 neonates with NEC. Age of NEC: not clear. Controls: 57 neonates without NEC. Fecal samples were collected at NEC onset (n = 53) and before NEC (n = 14). Data from Croix Rousse University Hospital: GA of 25–30 weeks. Cases: seven neonates with NEC. Controls: 11 neonates without NEC. Fecal samples were collected weekly during the first 30 days of life.	Shotgun metagenomic sequencing data sets from four different studies retrieved from publicly available repositories. Shotgun metagenomic sequencing. Taxonomic profiling of the retained reads was performed with the METAnnotatorX bioinformatics platform.	**Gut microbiota profiles:** The microbial profiles of metagenomics samples showed high interindividual variability. **NEC group:** had a higher abundance of *Escherichia coli* and *Enterococcus faecalis* were the main taxa. There was a preterm community state types (PT-CST)-specific increase in opportunistic pathogens, such as *E. faecalis*, *E. coli*, *Staphylococcus epidermidis*, *Clostridioides difficile*, *Ureaplasma parvum*, *Pseudomonas aeruginosa*, *Pseudomonas nosocomialis,* and members of the *Klebsiella* genus. There was a reduction in/absence of common early-infant gut commensals (*Actinomyces*, *Schaalia*, *Veillonella*, *Bacteroides*, and *Streptococcus*). *Clostridium neonatale* and *Clostridium perfringens* species could be potential biomarkers for the predictive early diagnosis of NEC. **Controls:** had a higher abundance of *Streptococcus agalactiae*, which was the dominant taxon in premature control subjects. Controls had a much lower relative abundance/absence of *K. pneumonia*, *Proteus mirabilis*, *Clostridium perfringens*, *Clostridium neonatale*, *Pantoea dispersa*, and *Staphylococcus aureus.* **Diversity:** There was a reduction in the gut microbial biodiversity in the NEC group. **Metabolic pathways**: in the NEC group, enzymes related to glycosylated protein degradation, i.e., α-fucosidase and sialidase, were almost entirely absent. α-fucosidase and sialidase were positively associated with members of the *Blautia*, *Cutibacterium,* and *Enterobacter* genera and negatively with *E. coli.* In the NEC group, bacterial tryptophan degradation pathways (tryptophanase enzymes (and indolepyruvate decarboxylase)) were increased. There was gastrointestinal DL-lactate accumulation among NEC patients.
14	He Y. (2021) [149] PMID: 34922582	Children’s Hospital of Chongqing Medical University, Chongqing, China. Study period: January 2015–October 2018. NEC stage: II–III.	Cases: 81 neonates with NEC (GA 31.0 (29.4–33.7) weeks); included 19 surgical NEC patients. Age of NEC: 15 (12–19). Controls: 81 neonates without NEC (GA 31.1 (29.3–33.2) weeks); included 19 surgical patients. Fecal samples were collected once the diagnosis of NEC was complete. SCFAs were measured in the fecal samples. Flow cytometry of T cells in ileum lamina propria was performed. The transcription of inflammatory cytokines by qRT-PCR. FMT to germ-free mice before NEC induction. Butyric acid administration.	16S ribosomal RNA gene (V3–V4 regions) sequencing The phylogenetic affiliation of each 16S rRNA gene sequence was analyzed using the Ribosomal Database Project.	**Gut microbiota profiles:** **NEC group:** had a higher abundance of *Pseudomonadota*, with reduced proportions of *Bacillota* and *Bacteroidota*. **Diversity:** There was decreased α-phylogenetic diversity in NEC patients. **SCFA measurement:** The levels of butyric acid were significantly lower in the NEC group. **Flow cytometry of T cells:** the proportion of Treg (Foxp3+) cells compared with T helper cells in the control group was significantly higher than that of the NEC group. **Cytokine expression:** The surgical NEC cases had significantly higher levels of IL-1β, IL-8, and TNF-α transcripts. NEC cases also had lower expression levels of IL-10 and TGF-β related to the induction and function of Treg cells. **Fecal-microbiota transplantation** of NEC patients contributed to NEC-like injury in mice. Mice had a decreased Treg/T helper cell ratio. **The NEC mice had a similar microbial composition as NEC patients had** a higher level of *Psudomonadota* but lower levels of *Bacillota*; the concentration of butyrate in the fecal samples was lower. Butyrate administration to mice increased the Treg/T helper cell ratio; mice had lower NEC scores. Butyrate administration did not influence the microbial composition.
15	Kaelin E. (2022) [163] PMID: 35449461	St. Louis Children’s Hospital, St. Louis, MO, USA. Study period: not clear. NEC stage: II or higher.	Preterm neonates with a birth weight ≤ 1500 g and GA < 27 weeks. Cases: nine neonates with NEC (GA of 25.5 (24.9–26.0) weeks). Age of NEC: not clear. Controls: 14 neonates without NEC (GA of 25.0 (23.1–25.4) weeks). Fecal samples were collected before NEC onset over the first 11 weeks of life.	Metagenomic sequencing of gut virome DNA.	There was high inter- and intra-individual variation in the infant gut virome. **NEC group:** A large proportion of the virome could not be assigned family-level taxonomy (unclassified viruses) (84.5%). Classifiable viral contigs included the bacteriophage families *Myoviridae*, *Podoviridae,* and *Siphoviridae*. There was high variability in virus family proportions at each time point and also within individuals over time. A total of 137 contigs were associated with the period 0–10 days before NEC (NEC-associated contigs). Of the NEC-associated contigs with at least five open reading frames (ORFs), 31.7% were predicted to have temperate lifestyles and 68.3% were predicted to be lytic. **Controls:** A large proportion of the virome was unclassified viruses (85.3%). The identified viral contigs belonged to bacteriophage families including *Myoviridae*, *Podoviridae,* and *Siphoviridae*. The relative abundances of bacteriophage and eukaryotic virus families varied between the control infants in each week of the study. **Diversity:** There was convergence towards reduced viral beta diversity over the 10 days before NEC onset, driven by specific viral signatures and viral–bacterial interactions. The bacterial beta diversity in NEC patients was stable in windows spanning the 25 days before NEC. The virome diversity in the control group varied between individuals.
16	Liu X.-C. (2022) [172] PMID: 36060739	Children’s Hospital of Chongqing Medical University, Chongqing, China. Study period: April 2021–October 2021. NEC stage: not clear.	Cases: 17 neonates with NEC (GA 30.5 ± 2.1 weeks). Controls: 17 neonates without NEC (GA 30.5 ± 1.9 weeks). Age of NEC: 30.2 ± 15.9. Fecal-sample collection: two time points (before NEC (7.0 ± 7.64 days before), and at the diagnosis of NEC). SCFAs were measured in feces.	16S ribosomal RNA gene (V3–V4 regions) sequencing Data processing with QIIME v1.9.1. Species classification was performed using silva138/16s_bacteria taxonomic data.	**Pre-NEC group vs. controls:***Pseudomonadota* increased while *Bacillota*, *Actinomycetota,* and *Bacteroidota* decreased (NS). *Clostridioides*, *Blautia,* and *Clostridium sensu stricto I* increased, while *unclassified_c_Bacilli*, *Lactobacillaceae*, and *Bifidobacterium* decreased at the genus level (*p* < 0.05). At the species level, *unclassified_g_Clostridioides*, *Streptococcus salivarius,* and *Rothia mucilaginosa* increased, while *unclassified_c_Bacilli*, *unclassified species of Lactobacillaceae*, and *Bifidobacterium animals* subsp. *lactis* decreased (*p* < 0.05). Acetic, propanoic, butyric, and isovaleric acids decreased. **NEC-group vs. controls:** *Bacillota* decreased. *Stenotrophomonas*, *Streptococcus,* and *Prevotella* increased at the genus level. Acetic, propanoic, butyric, and isobutyric acids decreased. **NEC vs. pre-NEC group:** *Pseudomonadota* and *Bacillota* decreased, while *Actinomycetota* and *Bacteroidota* increased (NS). At the genus level, *Faecalibacterium*, *Microbacterium,* and *Solobacterium* increased (*p* < 0.05). There were no differences in any of the SCFAs. **Diversity:** The alpha diversity (Ace, Chao1) was higher in controls than in pre-NEC patients, but the Simpson and Shannon indices showed no difference. There was no difference between NEC patients and controls.

This table shows the names of genera and species as given by the authors. Phylum, order, and family names were changed to the new nomenclature according to NCBI Taxonomy as required under the International Code of Nomenclature for Prokaryotes (ICNP). To date, some taxa have been reclassified or renamed. The correct names of these taxa are given in parentheses: *Bacteroides dorei* (*Phocaeicola dorei*).

## Data Availability

Not applicable.

## Abbreviations

2′-FL	2-fucosyllactose
3′-FL	3-fucosyllactose
3′-SL	3-sialyllactose
6′-SL	6-sialyllactose
BAX	Bcl-2-associated X protein
bFGF	basic fibroblast growth factor
CHD	congenital heart disease
DCDA	dichorionic diamniotic
DSS	dextran sulfate sodium
EGF	epidermal growth factor
eNOS	endothelial NO synthase
ET-1	endothelin-1
FGFR2	fibroblast growth factor receptor 2
F-Tr	GDP-fucose transporter
Fuc	fucose
FUK	fucokinase
FUT1	fucosyltransferase 1
FUT2	fucosyltransferase 2
FUT7	fucosyltransferase VII
FX	GDP-4-keto-6-deoxymannose 3,5- epimerase- 4-reductase
GA	gestational age
GDM	gestational diabetes mellitus
GFPP	GDP-β-l-fucose pyrophosphorylase
GL	genus level
GlcNAc	N-acetyl-glucosamine
GLUT1	glucose transporter 1
GMD	GDP-d-mannose-4,6-dehydratase
HIF	factors induced by hypoxia
HMO	human milk oligosaccharides
IBD	inflammatory bowel disease
IL-1β	interleukin-1β
IUGR	intrauterine growth retardation
Le^y^	Lewis Y
LPS	lipopolysaccharide
MCDA	monochorionic diamniotic twins
MUC1	mucin 1
MUC2	mucin-2
MUC5AC	mucin-5AC
NEC	necrotizing enterocolitis
NF-κB	nuclear factor kappa B
NO	nitric oxide
OTUs	operational taxonomic units
PAF	platelet-activating factor
PAI-1	plasminogen activator inhibitor-1
PDA	persistent ductus arteriosus
PE	pre-eclampsia
PHD3	HIF-prolyl hydroxylase 3
PI-CST	preterm community state types
PIGF	placental growth factor
PIH	pregnancy-induced hypertension
PL	phylum level
POFUT	protein O-fucosyltransferase
RIC	remote ischemic conditioning
ROS	reactive oxygen species
SCFAs	short-chain fatty acids
SCT	syncytiotrophoblast
sEng	soluble endoglin
sFlt-1	soluble fms-like tyrosine kinase-1
SIRT1	sirtuin 1
SL	species level
ST6GAL1	beta-galactosamide-alpha-2,6-sialyltransferase 1
TFF2	trefoil factor 2
TFF3	trefoil factor 3
TGF-β1	transforming growth factor beta 1
TLR4	toll-like receptor 4
TMAO	trimethylamine-N-oxide
TNF-α	tumor necrosis factor alpha
TSR	thrombospondin--type 1 repeats
UDP-galactose	uridine diphosphate galactose
UGT1	uridine diphosphate glucuronosyl transferase
VEGF	vascular endothelial growth factor
VEGFR	vascular endothelial growth factor receptor
VLBW	very low birth weight
WAZ	weight-for-age z-score
ZO-1	zonula occludens-1
α1,6-Fuc	α-1,6-fucosyltransferase

## References

[1] Meister A.L., Doheny K.K., Travagli R.A. (2020). Necrotizing enterocolitis: It’s not all in the gut. Exp. Biol. Med..

[2] Alsaied A., Islam N., Thalib L. (2020). Global incidence of necrotizing enterocolitis: A systematic review and meta-analysis. BMC Pediatr..

[3] Robinson J.R., Rellinger E.J., Hatch L.D., Weitkamp J.-H., Speck K.E., Danko M., Blakely M.L. (2017). Surgical necrotizing enterocolitis. Semin. Perinatol..

[4] van der Heide M., Mebius M.J., Bos A.F., Roofthooft M.T.R., Berger R.M.F., Hulscher J.B.F., Kooi E.M.W. (2020). Hypoxic/ischemic hits predispose to necrotizing enterocolitis in (near) term infants with congenital heart disease: A case control study. BMC Pediatr..

[5] Short S.S., Papillon S., Berel D., Ford H.R., Frykman P.K., Kawaguchi A. (2014). Late onset of necrotizing enterocolitis in the full-term infant is associated with increased mortality: Results from a two-center analysis. J. Pediatr. Surg..

[6] Verma R.P., Kota A., Shehata S. (2019). Necrotizing Enterocolitis. Pediatric Surgery, Flowcharts and Clinical Algorithms.

[7] Schnabl K.L., Aerde J.E.V., Thomson A.B., Clandinin M.T. (2008). Necrotizing enterocolitis: A multifactorial disease with no cure. World J. Gastroenterol..

[8] Ginglen J.G., Butki N. (2022). Necrotizing Enterocolitis. StatPearls.

[9] Cuna A., Sampath V. (2017). Genetic alterations in necrotizing enterocolitis. Semin. Perinatol..

[10] Magistris A.D., Marcialis M.A., Puddu M., Dessì A., Irmesi R., Coni E., Fanos V. (2016). Embryological development of the intestine and necrotizing enterocolitis. J. Pediatr. Neonat. Individ. Med..

[11] Sampah M.E.S., Hackam D.J. (2021). Prenatal immunity and influences on necrotizing enterocolitis and associated neonatal disorders. Front. Immunol..

[12] Kumbhare S.V., Patangia D.V.V., Patil R.H., Shouche Y.S., Patil N.P. (2019). Factors influencing the gut microbiome in children: From infancy to childhood. J. Biosci..

[13] Khoder-Agha F., Kietzmann T. (2021). The glyco-redox interplay: Principles and consequences on the role of reactive oxygen species during protein glycosylation. Redox Biol..

[14] Passaponti S., Pavone V., Cresti L., Ietta F. (2021). The expression and role of glycans at the feto-maternal interface in humans. Tissue Cell.

[15] Kononova S.V. (2017). How fucose of blood group glycotopes programs human gut microbiota. Biochemistry.

[16] Kudelka M.R., Stowell S.R., Cummings R.D., Neish A.S. (2020). Intestinal epithelial glycosylation in homeostasis and gut microbiota interactions in ibd. Nat. Rev. Gastroenterol. Hepatol..

[17] Hackam D.J., Sodhi C.P. (2018). Toll-like receptor-mediated intestinal inflammatory imbalance in the pathogenesis of necrotizing enterocolitis. Cell. Mol. Gastroenterol. Hepatol..

[18] Hunter C.J., De Plaen I.G. (2014). Inflammatory signaling in NEC: Role of NF-κB and cytokines. Pathophysiology.

[19] Nair J., Lakshminrusimha S. (2019). Role of NO and other vascular mediators in the etiopathogenesis of necrotizing enterocolitis. Front. Biosci. (School Ed.).

[20] Duci M., Frigo A.C., Visentin S., Verlato G., Gamba P., Fascetti-Leon F. (2019). Maternal and placental risk factors associated with the development of necrotizing enterocolitis (NEC) and its severity. J. Pediatr. Surg..

[21] Kamoji V.M., Dorling J.S., Manktelow B., Draper E.S., Field D.J. (2008). Antenatal umbilical doppler abnormalities: An independent risk factor for early onset neonatal necrotizing enterocolitis in premature infants. Acta Paediatr..

[22] Ahle M., Drott P., Elfvin A., Andersson R.E. (2018). Maternal, fetal and perinatal factors associated with necrotizing enterocolitis in Sweden. A national case-control study. PLoS ONE.

[23] Cao X., Zhang L., Jiang S., Li M., Yan C., Shen C., Yang Y., Lee S.K., Cao Y. (2022). Epidemiology of necrotizing enterocolitis in preterm infants in China: A multicenter cohort study from 2015 to 2018. J. Pediatr. Surg..

[24] Rose A.T., Patel R.M. (2018). A critical analysis of risk factors for necrotizing enterocolitis. Semin. Fetal Neonatal. Med..

[25] Wang K.-G., Chen C.-Y., Chen Y.-Y. (2009). The effects of absent or reversed end-diastolic umbilical artery doppler flow velocity. Taiwan J. Obstet. Gynecol..

[26] Wardinger J.E., Ambati S. (2022). Placental Insufficiency. StatPearls.

[27] Samuels N., van de Graaf R.A., de Jonge R.C.J., Reiss I.K.M., Vermeulen M.J. (2017). Risk factors for necrotizing enterocolitis in neonates: A systematic review of prognostic studies. BMC Pediatr..

[28] Aouache R., Biquard L., Vaiman D., Miralles F. (2018). Oxidative stress in preeclampsia and placental diseases. Int. J. Mol. Sci..

[29] Ree I.M.C., Smits-Wintjens V.E.H.J., Rijntjes-Jacobs E.G.J., Pelsma I.C.M., Steggerda S.J., Walther F.J., Lopriore E. (2014). Necrotizing Enterocolitis in Small-for-Gestational-Age Neonates: A Matched Case-Control Study. Neonatology.

[30] Yang C.-C., Tang P.-L., Liu P.-Y., Huang W.-C., Chen Y.-Y., Wang H.-P., Chang J.-T., Lin L.-T. (2018). Maternal pregnancy-induced hypertension increases subsequent neonatal necrotizing enterocolitis risk. Medicine.

[31] Samuel T.M., Sakwinska O., Makinen K., Burdge G.C., Godfrey K.M., Silva-Zolezzi I. (2019). Preterm Birth: A Narrative Review of the Current Evidence on Nutritional and Bioactive Solutions for Risk Reduction. Nutrients.

[32] Watson S.N., McElroy S.J. (2021). Potential prenatal origins of necrotizing enterocolitis. Gastroenterol. Clin. N. Am..

[33] Tan X., Zhou Y., Xu L., Zhang L., Wang J., Yang W. (2022). The predictors of necrotizing enterocolitis in newborns with low birth weight: A retrospective analysis. Medicine.

[34] Gephart S.M., Spitzer A.R., Effken J.A., Dodd E., Halpern M., McGrath J.M. (2014). Discrimination of GutCheck(NEC): A clinical risk index for necrotizing enterocolitis. J. Perinatol..

[35] Wang Y.-P., Zheng M.-Y., Xiao Y.-Y., Qu Y.-M., Wu H. (2022). Risk factors for necrotizing enterocolitis and establishment of prediction model of necrotizing enterocolitis in preterm infants. Zhongguo Dang Dai Er Ke Za Zhi (Chin. J. Contemp. Pediatr.).

[36] Berkhout D.J.C., Klaassen P., Niemarkt H.J., de Boode W.P., Cossey V., van Goudoever J.B., Hulzebos C.V., Andriessen P., van Kaam A.H., Kramer B.W. (2018). Risk Factors for Necrotizing Enterocolitis: A Prospective Multicenter Case-Control Study. Neonatology.

[37] Kordasz M., Racine M., Szavay P., Lehner M., Krebs T., Luckert C., Hau E.-M., Berger S., Kessler U. (2022). Risk factors for mortality in preterm infants with necrotizing enterocolitis: A retrospective multicenter analysis. Eur. J. Pediatr..

[38] Askie L.M., Darlow B.A., Davis P.G., Finer N., Stenson B., Vento M., Whyte R. (2017). Effects of targeting lower versus higher arterial oxygen saturations on death or disability in preterm infants. Cochrane Database Syst. Rev..

[39] Cotten C.M., Taylor S., Stoll B., Goldberg R.N., Hansen N.I., Sánchez P.J., Ambalavanan N., Benjamin D.K. (2009). Prolonged duration of initial empirical antibiotic treatment is associated with increased rates of necrotizing enterocolitis and death for extremely low birth weight infants. Pediatrics.

[40] Raba A.A., O’Sullivan A., Semberova J., Martin A., Miletin J. (2019). Are antibiotics a risk factor for the development of necrotizing enterocolitis—Case-control retrospective study. Eur. J. Pediatr..

[41] Zwittink R.D., van Zoeren-Grobben D., Martin R., van Lingen R.A., Groot Jebbink L.J., Boeren S., Renes I.B., van Elburg R.M., Belzer C., Knol J. (2017). Metaproteomics reveals functional differences in intestinal microbiota development of preterm infants. Mol. Cell. Proteomics.

[42] Lemme-Dumit J.M., Song Y., Lwin H.W., Hernandez-Chavez C., Sundararajan S., Viscardi R.M., Ravel J., Pasetti M.F., Ma B. (2022). Altered gut microbiome and fecal immune phenotype in early preterm infants with leaky gut. Front. Immunol..

[43] Koren O., Goodrich J.K., Cullender T.C., Spor A., Laitinen K., Kling Bäckhed H., Gonzalez A., Werner J.J., Angenent L.T., Knight R. (2012). Host remodeling of the gut microbiome and metabolic changes during pregnancy. Cell.

[44] Dahl C., Stanislawski M., Iszatt N., Mandal S., Lozupone C., Clemente J.C., Knight R., Stigum H., Eggesbø M. (2017). Gut microbiome of mothers delivering prematurely shows reduced diversity and lower relative abundance of *Bifidobacterium* and *Streptococcus*. PLoS ONE.

[45] Wang Z.-L., An Y., He Y., Hu X.-Y., Guo L., Li Q.-Y., Liu L., Li L.-Q. (2020). Risk factors of necrotizing enterocolitis in neonates with sepsis: A retrospective case-control study. Int. J. Immunopathol. Pharmacol..

[46] Bäckhed F., Roswall J., Peng Y., Feng Q., Jia H., Kovatcheva-Datchary P., Li Y., Xia Y., Xie H., Zhong H. (2015). Dynamics and stabilization of the human gut microbiome during the first year of life. Cell Host Microbe.

[47] Heida F.H., Kooi E.M.W., Wagner J., Nguyen T.-Y., Hulscher J.B.F., van Zoonen A.G.J.F., Bos A.F., Harmsen H.J.M., de Goffau M.C. (2021). Weight shapes the intestinal microbiome in preterm infants: Results of a prospective observational study. BMC Microbiol..

[48] Russell J.T., Lauren Ruoss J., de la Cruz D., Li N., Bazacliu C., Patton L., McKinley K.L., Garrett T.J., Polin R.A., Triplett E.W. (2021). Antibiotics and the developing intestinal microbiome, metabolome and inflammatory environment in a randomized trial of preterm infants. Sci. Rep..

[49] Bowker R.M., Yan X., De Plaen I.G. (2018). Intestinal microcirculation and necrotizing enterocolitis: The vascular endothelial growth factor system. Semin. Fetal. Neonatal. Med..

[50] Surmeli Onay O., Korkmaz A., Yigit S., Yurdakok M. (2020). Hypoxic-Ischemic Enterocolitis: A proposal of a new terminology for early NEC or NEC-like disease in preterm infants, a single-center prospective observational study. Eur. J. Pediatr..

[51] Taylor C.T., Colgan S.P. (2017). Regulation of immunity and inflammation by hypoxia in immunological niches. Nat. Rev. Immunol..

[52] Sagrillo-Fagundes L., Laurent L., Bienvenue-Pariseault J., Vaillancourt C. (2018). In vitro induction of hypoxia/reoxygenation on placental cells: A suitable model for understanding placental diseases. Methods Mol. Biol..

[53] Lien Y.-C., Zhang Z., Cheng Y., Polyak E., Sillers L., Falk M.J., Ischiropoulos H., Parry S., Simmons R.A. (2021). Human placental transcriptome reveals critical alterations in inflammation and energy metabolism with fetal sex differences in spontaneous preterm birth. Int. J. Mol. Sci..

[54] Konjar Š., Pavšič M., Veldhoen M. (2021). Regulation of oxygen homeostasis at the intestinal epithelial barrier site. Int. J. Mol. Sci..

[55] Belo A.I., van Vliet S.J., Maus A., Laan L.C., Nauta T.D., Koolwijk P., Tefsen B., van Die I. (2015). Hypoxia inducible factor 1α down regulates cell surface expression of α1,2-fucosylated glycans in human pancreatic adenocarcinoma cells. FEBS Lett..

[56] McCracken S.A., Seeho S.K.M., Carrodus T., Park J.H., Woodland N., Gallery E.D.M., Morris J.M., Ashton A.W. (2022). Dysregulation of oxygen sensing/response pathways in pregnancies complicated by idiopathic intrauterine growth restriction and early-onset preeclampsia. Int. J. Mol. Sci..

[57] Blois S.M., Prince P.D., Borowski S., Galleano M., Barrientos G. (2021). Placental glycoredox dysregulation associated with disease progression in an animal model of superimposed preeclampsia. Cells.

[58] Kulikova G.V., Ziganshina M.M., Shchegolev A.I., Sukhikh G.T. (2021). Comparative characteristics of the expression of fucosylated glycans and morphometric parameters of terminal placental villi depending on the severity of preeclampsia. Bull. Exp. Biol. Med..

[59] Ahmadian E., Rahbar Saadat Y., Hosseiniyan Khatibi S.M., Nariman-Saleh-Fam Z., Bastami M., Zununi Vahed F., Ardalan M., Zununi Vahed S. (2020). Pre-Eclampsia: Microbiota possibly playing a role. Pharmacol. Res..

[60] Tsou P.-S., Ruth J.H., Campbell P.L., Isozaki T., Lee S., Marotte H., Domino S.E., Koch A.E., Amin M.A. (2013). A Novel role for inducible Fut2 in angiogenesis. Angiogenesis.

[61] Chen Y., Koike Y., Chi L., Ahmed A., Miyake H., Li B., Lee C., Delgado-Olguín P., Pierro A. (2019). Formula feeding and immature gut microcirculation promote intestinal hypoxia, leading to necrotizing enterocolitis. Dis. Model. Mech..

[62] Koike Y., Li B., Ganji N., Zhu H., Miyake H., Chen Y., Lee C., Janssen Lok M., Zozaya C., Lau E. (2020). Remote Ischemic Conditioning Counteracts the Intestinal Damage of Necrotizing Enterocolitis by Improving Intestinal Microcirculation. Nat. Commun..

[63] Henderickx J.G.E., Zwittink R.D., Renes I.B., van Lingen R.A., van Zoeren-Grobben D., Jebbink L.J.G., Boeren S., van Elburg R.M., Knol J., Belzer C. (2021). Maturation of the Preterm Gastrointestinal Tract Can Be Defined by Host and Microbial Markers for Digestion and Barrier Defense. Sci. Rep..

[64] Irons E.E., Cortes Gomez E., Andersen V.L., Lau J.T.Y. (2022). Bacterial colonization and TH17 immunity are shaped by intestinal sialylation in neonatal mice. Glycobiology.

[65] Sodhi C.P., Neal M.D., Siggers R., Sho S., Ma C., Branca M.F., Prindle T., Russo A.M., Afrazi A., Good M. (2012). Intestinal epithelial Toll-like receptor 4 regulates goblet cell development and is required for necrotizing enterocolitis in mice. Gastroenterology.

[66] Iijima J., Kobayashi S., Kitazume S., Kizuka Y., Fujinawa R., Korekane H., Shibata T., Saitoh S.I., Akashi-Takamura S., Miyake K. (2017). Core fucose is critical for CD14-dependent Toll-like receptor 4 signals. Glycobiology.

[67] Sodhi C.P., Wipf P., Yamaguchi Y., Fulton W.B., Kovler M., Niño D.F., Zhou Q., Banfield E., Werts A.D., Ladd M.R. (2021). The human milk oligosaccharides 2′-fucosyllactose and 6′-sialyllactose protect against the development of necrotizing enterocolitis by inhibiting toll-like receptor 4 signaling. Pediatr. Res..

[68] Griffiths V., Al Assaf N., Khan R. (2021). Review of claudin proteins as potential biomarkers for necrotizing enterocolitis. Ir. J. Med. Sci..

[69] Bein A., Eventov-Friedman S., Arbell D., Schwartz B. (2018). Intestinal tight junctions are severely altered in NEC preterm neonates. Pediatr. Neonatol..

[70] Bai M., Lu C., An L., Gao Q., Xie W., Miao F., Chen X., Pan Y., Wang Q. (2020). SIRT1 relieves necrotizing enterocolitis through inactivation of hypoxia-inducible factor (HIF)-1a. Cell Cycle.

[71] Högberg N., Stenbäck A., Carlsson P.-O., Wanders A., Lilja H.E. (2013). Genes regulating tight junctions and cell adhesion are altered in early experimental necrotizing enterocolitis. J. Pediatr. Surg..

[72] Nolan L.S., Rimer J.M., Good M. (2020). The Role of Human Milk Oligosaccharides and Probiotics on the Neonatal Microbiome and Risk of Necrotizing Enterocolitis: A Narrative Review. Nutrients.

[73] Yan X., Managlia E., Tan X.-D., De Plaen I.G. (2019). Prenatal inflammation impairs intestinal microvascular development through a TNF-dependent mechanism and predisposes newborn mice to necrotizing enterocolitis. Am. J. Physiol. Gastrointest. Liver. Physiol..

[74] Kunz C., Meyer C., Collado M.C., Geiger L., García-Mantrana I., Bertua-Ríos B., Martínez-Costa C., Borsch C., Rudloff S. (2017). Influence of gestational age, secretor, and Lewis blood group status on the oligosaccharide content of human milk. J. Pediatr. Gastroenterol. Nutr..

[75] Urashima T., Taufik E., Fukuda K., Asakuma S. (2013). Recent advances in studies on milk oligosaccharides of cows and other domestic farm animals. Biosci. Biotechnol. Biochem..

[76] Mollicone R., Cailleau A., Oriol R. (1995). Molecular genetics of H, Se, Lewis and other fucosyltransferase genes. Transfus. Clin. Biol..

[77] Morrow A.L., Meinzen-Derr J., Huang P., Schibler K.R., Cahill T., Keddache M., Kallapur S.G., Newburg D.S., Tabangin M., Warner B.B. (2011). Fucosyltransferase 2 non-secretor and low secretor status predicts severe outcomes in premature infants. J. Pediatr..

[78] Ye Q., Yu J. (2021). A study on fucosyltransferase 2 gene polymorphism and secretion status related to neonatal necrotizing enterocolitis. J. Healthc. Eng..

[79] She X., Du H., Yi C., He Y., Ai Q., Yu J. (2021). The decrease of fucosylation in intestinal epithelium is related to the development of necrotizing enterocolitis. Mol. Immunol..

[80] Demmert M., Schaper A., Pagel J., Gebauer C., Emeis M., Heitmann F., Kribs A., Siegel J., Müller D., Keller-Wackerbauer A. (2015). FUT 2 polymorphism and outcome in very-low-birth-weight infants. Pediatr. Res..

[81] Nuzzi G., Trambusti I., DI Cicco M.E., Peroni D.G. (2021). Breast milk: More than just nutrition!. Minerva Pediatr..

[82] Sánchez C., Franco L., Regal P., Lamas A., Cepeda A., Fente C. (2021). Breast Milk: A Source of Functional Compounds with Potential Application in Nutrition and Therapy. Nutrients.

[83] Kononova S., Litvinova E., Vakhitov T., Skalinskaya M., Sitkin S. (2021). Acceptive immunity: The role of fucosylated glycans in human host–microbiome interactions. Int. J. Mol. Sci..

[84] Cheema A.S., Trevenen M.L., Turlach B.A., Furst A.J., Roman A.S., Bode L., Gridneva Z., Lai C.T., Stinson L.F., Payne M.S. (2022). Exclusively breastfed infant microbiota develops over time and is associated with human milk oligosaccharide intakes. Int. J. Mol. Sci..

[85] Liu F., Yan J., Wang X., Wang C., Chen L., Li Y., Chen J., Guo H. (2021). Maternal fucosyltransferase 2 status associates with the profiles of human milk oligosaccharides and the fecal microbiota composition of breastfed infants. J. Agric. Food Chem..

[86] Cabrera-Rubio R., Kunz C., Rudloff S., García-Mantrana I., Crehuá-Gaudiza E., Martínez-Costa C., Collado M.C. (2019). Association of maternal secretor status and human milk oligosaccharides with milk microbiota: An observational pilot study. J. Pediatr. Gastroenterol. Nutr..

[87] Lewis Z.T., Totten S.M., Smilowitz J.T., Popovic M., Parker E., Lemay D.G., Van Tassell M.L., Miller M.J., Jin Y.S., German J.B. (2015). Maternal fucosyltransferase 2 status affects the gut bifidobacterial communities of breastfed infants. Microbiome.

[88] Caldwell J., Matson A., Mosha M., Hagadorn J.I., Moore J., Brownell E. (2021). Maternal H-antigen secretor status is an early biomarker for potential preterm delivery. J. Perinatol..

[89] Li B., Wu R.Y., Horne R.G., Ahmed A., Lee D., Robinson S.C., Zhu H., Lee C., Cadete M., Johnson-Henry K.C. (2020). Human Milk Oligosaccharides Protect against Necrotizing Enterocolitis by Activating Intestinal Cell Differentiation. Mol. Nutr. Food Res..

[90] Becker D.J., Lowe J.B. (2003). Fucose: Biosynthesis and biological function in mammals. Glycobiology.

[91] Keeley T.S., Yang S., Lau E. (2019). The diverse contributions of fucose linkages in cancer. Cancers.

[92] Honas B.J., Glassman U.M., Wiese T.J. (2009). Enzymatic activity of alpha-L-fucosidase and L-fucokinase across vertebrate animal species. Comp. Biochem. Physiol. B Biochem. Mol. Biol..

[93] Sosicka P., Ng B.G., Wong M., Xia Z.J., Scott D., Lebrilla C.B., Freeze H.H. (2020). Novel insights into the fucose metabolism–challenging the old dogma. FASEB J..

[94] Park D., Ryu K.S., Choi D., Kwak J., Park C. (2007). Characterization and role of fucose mutarotase in mammalian cells. Glycobiology.

[95] Skurska E., Szulc B., Maszczak-Seneczko D., Wiktor M., Wiertelak W., Makowiecka A., Olczak M. (2022). Incorporation of fucose into glycans independent of the GDP-fucose transporter SLC35C1 preferentially utilizes salvaged over *de novo* GDP-fucose. J. Biol. Chem..

[96] Xu Y.X., Ma A., Liu L. (2013). Transforming growth factor β signaling upregulates the expression of human GDP-fucose transporter by activating transcription factor Sp1. PLoS ONE.

[97] Li Y., Jiang Y., Zhang L., Qian W., Hou X., Lin R. (2021). Exogenous l-fucose protects the intestinal mucosal barrier depending on upregulation of FUT2-mediated fucosylation of intestinal epithelial cells. FASEB J..

[98] De Leoz M.L., Gaerlan S.C., Strum J.S., Dimapasoc L.M., Mirmiran M., Tancredi D.J., Smilowitz J.T., Kalanetra K.M., Mills D.A., German J.B. (2012). Lacto-N-tetraose, fucosylation, and secretor status are highly variable in human milk oligosaccharides from women delivering preterm. J. Proteome Res..

[99] Jilling T., Ambalavanan N., Cotton C.M., Martin C.A., Maheshwari A., Schibler K., Levy J., Page G.P. (2018). Surgical necrotizing enterocolitis in extremely premature neonates is associated with genetic variations in an intergenic region of chromosome 8. Pediatr. Res..

[100] Thomson P., Medina D.A., Garrido D. (2018). Human milk oligosaccharides and infant gut bifidobacteria: Molecular strategies for their utilization. Food Microbiol..

[101] Sakanaka M., Gotoh A., Yoshida K., Odamaki T., Koguchi H., Xiao J.Z., Kitaoka M., Katayama T. (2019). Varied Pathways of Infant Gut-Associated *Bifidobacterium* to Assimilate Human Milk Oligosaccharides: Prevalence of the Gene Set and its Correlation with Bifidobacteria-Rich Microbiota Formation. Nutrients.

[102] Rodríguez-Díaz J., Rubio-del-Campo A., Yebra M.J. (2012). *Lactobacillus casei* ferments the N-Acetylglucosamine moiety of fucosyl-α-1,3-N-acetylglucosamine and excretes L-fucose. Appl. Environ. Microbiol..

[103] Hiltunen H., Collado M.C., Ollila H., Kola T., Tölkkö S., Isolauri E., Salminen S., Rautava S. (2022). Spontaneous preterm delivery is reflected in both early neonatal and maternal gut microbiota. Pediatr. Res..

[104] Ferretti P., Pasolli E., Tett A., Asnicar F., Gorfer V., Fedi S., Armanini F., Truong D.T., Manara S., Zolfo M. (2018). Mother-to-infant microbial transmission from different body sites shapes the developing infant gut microbiome. Cell Host Microbe.

[105] Yang H., Guo R., Li S., Liang F., Tian C., Zhao X., Long Y., Liu F., Jiang M., Zhang Y. (2020). Systematic analysis of gut microbiota in pregnant women and its correlations with individual heterogeneity. Npj Biofilms Microbiomes.

[106] Kumar H., Wacklin P., Nakphaichit M., Loyttyniemi E., Chowdhury S., Shouche Y., Mättö J., Isolauri E., Salminen S. (2015). Secretor status is strongly associated with microbial alterations observed during pregnancy. PLoS ONE.

[107] Liu J., Yang H., Yin Z., Jiang X., Zhong H., Qiu D., Zhu F., Li R. (2017). Remodeling of the gut microbiota and structural shifts in Preeclampsia patients in South China. Eur. J. Clin. Microbiol. Infect. Dis..

[108] Lv L.-J., Li S.-H., Li S.-C., Zhong Z.-C., Duan H.-L., Tian C., Li H., He W., Chen M.-C., He T.-W. (2019). Early-onset preeclampsia is associated with gut microbial alterations in antepartum and postpartum women. Front. Cell. Infect. Microbiol..

[109] DiGiulio D.B., Callahan B.J., McMurdie P.J., Costello E.K., Lyell D.J., Robaczewska A., Sun C.L., Goltsman D.S.A., Wong R.J., Shaw G. (2015). Temporal and spatial variation of the human microbiota during pregnancy. Proc. Natl. Acad. Sci. USA.

[110] Callahan B.J., DiGiulio D.B., Goltsman D.S.A., Sun C.L., Costello E.K., Jeganathan P., Biggio J.R., Wong R.J., Druzin M.L., Shaw G.M. (2017). Replication and refinement of a vaginal microbial signature of preterm birth in two racially distinct cohorts of US women. Proc. Natl. Acad. Sci. USA.

[111] Zakaria Z.Z., Al-Rumaihi S., Al-Absi R.S., Farah H., Elamin M., Nader R., Bouabidi S., Suleiman S.E., Nasr S., Al-Asmakh M. (2022). Physiological changes and interactions between microbiome and the host during pregnancy. Front. Cell. Infect. Microbiol..

[112] Gough E.K., Edens T.J., Geum H.M., Baharmand I., Gill S.K., Robertson R.C., Mutasa K., Ntozini R., Smith L.E., Chasekwa B. (2021). Maternal fecal microbiome predicts gestational age, birth weight and neonatal growth in rural Zimbabwe. eBioMedicine.

[113] Li D., Huang Y., Sadykova A., Zheng W., Lin L., Jin C., Zhong W., Liao C., Pan S. (2021). Composition of the microbial communities at different body sites in women with preterm birth and their newborns. Med. Microecol..

[114] Wang J., Shi Z.-H., Yang J., Wei Y., Wang X.-Y., Zhao Y.-Y. (2020). Gut microbiota dysbiosis in preeclampsia patients in the second and third trimesters. Chin. Med. J..

[115] Qing W., Shi Y., Zhou H., Chen M. (2021). Gut microbiota dysbiosis in patients with preeclampsia: A systematic review. Med. Microecol..

[116] Ishimwe J.A. (2021). Maternal microbiome in preeclampsia pathophysiology and implications on offspring health. Physiol. Rep..

[117] Miao T., Yu Y., Sun J., Ma A., Yu J., Cui M., Yang L., Wang H. (2021). Decrease in abundance of bacteria of the genus *Bifidobacterium* in gut microbiota may be related to pre-eclampsia progression in women from East China. Food. Nutr. Res..

[118] Chen X., Li P., Liu M., Zheng H., He Y., Chen M.-X., Tang W., Yue X., Huang Y., Zhuang L. (2020). Gut dysbiosis induces the development of pre-eclampsia through bacterial translocation. Gut.

[119] Wang J., Gu X., Yang J., Wei Y., Zhao Y. (2019). Gut microbiota dysbiosis and increased plasma LPS and TMAO levels in patients with preeclampsia. Front. Cell. Infect. Microbiol..

[120] Gomez-Arango L.F., Barrett H.L., McIntyre H.D., Callaway L.K., Morrison M., Dekker Nitert M. (2016). Increased systolic and diastolic blood pressure is associated with altered gut microbiota composition and butyrate production in early pregnancy. Hypertension.

[121] Huang L., Cai M., Li L., Zhang X., Xu Y., Xiao J., Huang Q., Luo G., Zeng Z., Jin C. (2021). Gut microbiota changes in preeclampsia, abnormal placental growth and healthy pregnant women. BMC Microbiol..

[122] Susic D.F., Wang L., Roberts L.M., Bai M., Gia A., McGovern E., Jiang X.-T., Davis G.K., El-Omar E., Henry A. (2022). The P4 study: Postpartum maternal and infant faecal microbiome 6 months after hypertensive versus normotensive pregnancy. Front. Cell. Infect. Microbiol..

[123] Jin J., Gao L., Zou X., Zhang Y., Zheng Z., Zhang X., Li J., Tian Z., Wang X., Gu J. (2022). Gut dysbiosis promotes preeclampsia by regulating macrophages and trophoblasts. Circ. Res..

[124] Li P., Wang H., Guo L., Gou X., Chen G., Lin D., Fan D., Guo X., Liu Z. (2022). Association between gut microbiota and preeclampsia-eclampsia: A two-sample mendelian randomization study. BMC Med..

[125] Lv L.-J., Li S.-H., Wen J.-Y., Wang G.-Y., Li H., He T.-W., Lv Q.-B., Xiao M.-C., Duan H.-L., Chen M.-C. (2022). Deep metagenomic characterization of gut microbial community and function in preeclampsia. Front. Cell. Infect. Microbiol..

[126] Lin H., Chen J., Ma S., An R., Li X., Tan H. (2022). The Association between Gut Microbiome and Pregnancy-Induced Hypertension: A Nested Case–Control Study. Nutrients.

[127] Tu X., Duan C., Lin B., Li K., Gao J., Yan H., Wang K., Zhao Z. (2022). Characteristics of the gut microbiota in pregnant women with fetal growth restriction. BMC Pregnancy Childbirth.

[128] Yang J., Hou L., Wang J., Xiao L., Zhang J., Yin N., Yao S., Cheng K., Zhang W., Shi Z. (2022). Unfavourable intrauterine environment contributes to abnormal gut microbiome and metabolome in twins. Gut.

[129] Sun Y., Li L., Song J., Mao W., Xiao K., Jiang C. (2021). Intrauterine hypoxia changed the colonization of the gut microbiota in newborn rats. Front. Pediatr..

[130] Tarracchini C., Milani C., Longhi G., Fontana F., Mancabelli L., Pintus R., Lugli G.A., Alessandri G., Anzalone R., Viappiani A. (2021). Unraveling the microbiome of necrotizing enterocolitis: Insights in novel microbial and metabolomic biomarkers. Microbiol. Spectr..

[131] Arboleya S., Rios-Covian D., Maillard F., Langella P., Gueimonde M., Martín R. (2022). Preterm delivery: Microbial dysbiosis, gut inflammation and hyperpermeability. Front. Microbiol..

[132] Hiltunen H., Hanani H., Luoto R., Turjeman S., Ziv O., Isolauri E., Salminen S., Koren O., Rautava S. (2021). Preterm infant meconium microbiota transplant induces growth failure, inflammatory activation, and metabolic disturbances in germ-free mice. Cell. Rep. Med..

[133] Rodríguez-Benítez M.V., Gámez-Belmonte R., Gil-Campos M., Hernández-Chirlaque C., Bouzas P.R., Sánchez de Medina F., Martínez-Augustin O. (2021). Premature birth infants present elevated inflammatory markers in the meconium. Front. Pediatr..

[134] Heida F.H., van Zoonen A.G.J.F., Hulscher J.B.F., Te Kiefte B.J.C., Wessels R., Kooi E.M.W., Bos A.F., Harmsen H.J.M., de Goffau M.C. (2016). A Necrotizing Enterocolitis-Associated Gut Microbiota Is Present in the Meconium: Results of a Prospective Study. Clin. Infect. Dis..

[135] Klopp J., Ferretti P., Meyer C.U., Hilbert K., Haiß A., Marißen J., Henneke P., Hudalla H., Pirr S., Viemann D. (2022). Meconium microbiome of very preterm infants across Germany. mSphere.

[136] Wandro S., Osborne S., Enriquez C., Bixby C., Arrieta A., Whiteson K. (2018). The microbiome and metabolome of preterm infant stool are personalized and not driven by health outcomes, including necrotizing enterocolitis and late-onset sepsis. mSphere.

[137] Chang H.-Y., Chiang Chiau J.-S., Ho Y.-H., Chang J.-H., Tsai K.-N., Liu C.-Y., Hsu C.-H., Lin C.-Y., Ko M.H.-J., Lee H.-C. (2021). Impact of early empiric antibiotic regimens on the gut microbiota in very low birth weight preterm infants: An observational study. Front. Pediatr..

[138] Zwittink R.D., van Zoeren-Grobben D., Renes I.B., van Lingen R.A., Norbruis O.F., Martin R., Groot Jebbink L.J., Knol J., Belzer C. (2020). Dynamics of the bacterial gut microbiota in preterm and term infants after intravenous amoxicillin/ceftazidime treatment. BMC Pediatr..

[139] La Rosa P.S., Warner B.B., Zhou Y., Weinstock G.M., Sodergren E., Hall-Moore C.M., Stevens H.J., Bennett W.E., Shaikh N., Linneman L.A. (2014). Patterned progression of bacterial populations in the premature infant gut. Proc. Natl. Acad. Sci. USA.

[140] Moles L., Gómez M., Heilig H., Bustos G., Fuentes S., de Vos W., Fernández L., Rodríguez J.M., Jiménez E. (2013). Bacterial diversity in meconium of preterm neonates and evolution of their fecal microbiota during the first month of life. PLoS ONE.

[141] Aguilar-Lopez M., Dinsmoor A.M., Ho T.T.B., Donovan S.M. (2021). A Systematic Review of the Factors influencing microbial colonization of the preterm infant gut. Gut Microbes.

[142] Yu Y., Lu L., Sun J., Petrof E.O., Claud E.C. (2016). Preterm infant gut microbiota affects intestinal epithelial development in a humanized microbiome gnotobiotic mouse model. Am. J. Physiol. Gastrointest. Liver Physiol..

[143] Ho T.T.B., Groer M.W., Kane B., Yee A.L., Torres B.A., Gilbert J.A., Maheshwari A. (2018). Dichotomous development of the gut microbiome in preterm infants. Microbiome.

[144] Rohmer L., Hocquet D., Miller S.I. (2011). Are pathogenic bacteria just looking for food? Metabolism and microbial pathogenesis. Trends Microbiol..

[145] Watkins E.R., Maiden M.C., Gupta S. (2016). Metabolic competition as a driver of bacterial population structure. Future Microbiol..

[146] Hosny M., Baptiste E., Levasseur A., La Scola B. (2019). Molecular epidemiology of clostridium neonatale and its relationship with the occurrence of necrotizing enterocolitis in preterm neonates. New Microbes New Infect..

[147] Petrova N.A., Kaplina A.V., Khavkin A.I., Pervunina T.M., Komlichenko E.V., Nikiforov V.G., Sitkin S.I. (2021). Necrotizing enterocolitis: Current concepts of etiopathogenesis with an emphasis on microbiome and metabolomics. Vopr. Prakt. Pediatr. (Clin. Pract. Pediatr.).

[148] Dobbler P.T., Procianoy R.S., Mai V., Silveira R.C., Corso A.L., Rojas B.S., Roesch L.F.W. (2017). Low microbial diversity and abnormal microbial succession is associated with necrotizing enterocolitis in preterm infants. Front. Microbiol..

[149] He Y., Du W., Xiao S., Zeng B., She X., Liu D., Du H., Li L., Li F., Ai Q. (2021). Colonization of fecal microbiota from patients with neonatal necrotizing enterocolitis exacerbates intestinal injury in germfree mice subjected to necrotizing enterocolitis-induction protocol via alterations in butyrate and regulatory T cells. J. Transl. Med..

[150] Lindberg T.P., Caimano M.J., Hagadorn J.I., Bennett E.M., Maas K., Brownell E.A., Matson A.P. (2020). Preterm infant gut microbial patterns related to the development of necrotizing enterocolitis. J. Matern. Fetal Neonatal Med..

[151] Olm M.R., Bhattacharya N., Crits-Christoph A., Firek B.A., Baker R., Song Y.S., Morowitz M.J., Banfield J.F. (2019). Necrotizing enterocolitis is preceded by increased gut bacterial replication, *Klebsiella*, and fimbriae-encoding bacteria. Sci. Adv..

[152] Warner B.B., Deych E., Zhou Y., Hall-Moore C., Weinstock G.M., Sodergren E., Shaikh N., Hoffmann J.A., Linneman L.A., Hamvas A. (2016). Gut bacteria dysbiosis and necrotising enterocolitis in very low birthweight infants: A prospective case-control study. Lancet.

[153] Pammi M., Cope J., Tarr P.I., Warner B.B., Morrow A.L., Mai V., Gregory K.E., Kroll J.S., McMurtry V., Ferris M.J. (2017). Intestinal dysbiosis in preterm infants preceding necrotizing enterocolitis: A systematic review and meta-analysis. Microbiome.

[154] Ward D.V., Scholz M., Zolfo M., Taft D.H., Schibler K.R., Tett A., Segata N., Morrow A.L. (2016). Metagenomic sequencing with strain-level resolution implicates uropathogenic *E. coli* in necrotizing enterocolitis and mortality in preterm infants. Cell. Rep..

[155] Fahey R.C., Brown W.C., Adams W.B., Worsham M.B. (1978). Occurrence of glutathione in bacteria. J. Bacteriol..

[156] Romano-Keeler J., Shilts M.H., Tovchigrechko A., Wang C., Brucker R.M., Moore D.J., Fonnesbeck C., Meng S., Correa H., Lovvorn H.N. (2018). Distinct mucosal microbial communities in infants with surgical necrotizing enterocolitis correlate with age and antibiotic exposure. PLoS ONE.

[157] Rozé J.-C., Ancel P.-Y., Lepage P., Martin-Marchand L., Al Nabhani Z., Delannoy J., Picaud J.-C., Lapillonne A., Aires J., Durox M. (2017). Nutritional strategies and gut microbiota composition as risk factors for necrotizing enterocolitis in very-preterm infants. Am. J. Clin. Nutr..

[158] Fu C.-Y., Li L.-Q., Yang T., She X., Ai Q., Wang Z.-L. (2020). Autoinducer-2 may be a new biomarker for monitoring neonatal necrotizing enterocolitis. Front. Cell. Infect. Microbiol..

[159] Kaiko G.E., Ryu S.H., Koues O.I., Collins P.L., Solnica-Krezel L., Pearce E.J., Pearce E.L., Oltz E.M., Stappenbeck T.S. (2016). The colonic crypt protects stem cells from microbiota-derived metabolites. Cell.

[160] Salvi P.S., Cowles R.A. (2021). Butyrate and the intestinal epithelium: Modulation of proliferation and inflammation in homeostasis and disease. Cells.

[161] Zhou Y., Shan G., Sodergren E., Weinstock G., Walker W.A., Gregory K.E. (2015). longitudinal analysis of the premature infant intestinal microbiome prior to necrotizing enterocolitis: A case-control study. PLoS ONE.

[162] Fu X., Li S., Jiang Y., Hu X., Wu H. (2021). Necrotizing enterocolitis and intestinal microbiota: The timing of disease and combined effects of multiple species. Front. Pediatr..

[163] Kaelin E.A., Rodriguez C., Hall-Moore C., Hoffmann J.A., Linneman L.A., Ndao I.M., Warner B.B., Tarr P.I., Holtz L.R., Lim E.S. (2022). Longitudinal gut virome analysis identifies specific viral signatures that precede necrotizing enterocolitis onset in preterm infants. Nat. Microbiol..

[164] Jayasinghe T.N., Vatanen T., Chiavaroli V., Jayan S., McKenzie E.J., Adriaenssens E., Derraik J.G.B., Ekblad C., Schierding W., Battin M.R. (2020). Differences in compositions of gut bacterial populations and bacteriophages in 5–11 year-olds born preterm compared to full term. Front. Cell. Infect. Microbiol..

[165] Lin H., Guo Q., Ran Y., Lin L., Chen P., He J., Chen Y., Wen J. (2021). Multiomics Study Reveals *Enterococcus* and *Subdoligranulum* Are Beneficial to Necrotizing Enterocolitis. Front. Microbiol..

[166] Federici S., Kredo-Russo S., Valdés-Mas R., Kviatcovsky D., Weinstock E., Matiuhin Y., Silberberg Y., Atarashi K., Furuichi M., Oka A. (2022). Targeted suppression of human IBD-associated gut microbiota commensals by phage consortia for treatment of intestinal inflammation. Cell.

[167] Renwick V.L., Stewart C.J. (2022). Exploring functional metabolites in preterm infants. Acta Paediatr..

[168] Morrow A.L., Lagomarcino A.J., Schibler K.R., Taft D.H., Yu Z., Wang B., Altaye M., Wagner M., Gevers D., Ward D.V. (2013). Early microbial and metabolomic signatures predict later onset of necrotizing enterocolitis in preterm infants. Microbiome.

[169] Thomaidou A., Chatziioannou A.C., Deda O., Benaki D., Gika H., Mikros E., Agakidis C., Raikos N., Theodoridis G., Sarafidis K. (2019). A Pilot case-control study of urine metabolomics in preterm neonates with necrotizing enterocolitis. J. Chromatogr. B Analyt. Technol. Biomed. Life Sci..

[170] Stewart C.J., Embleton N.D., Marrs E.C.L., Smith D.P., Nelson A., Abdulkadir B., Skeath T., Petrosino J.F., Perry J.D., Berrington J.E. (2016). Temporal bacterial and metabolic development of the preterm gut reveals specific signatures in health and disease. Microbiome.

[171] Brehin C., Dubois D., Dicky O., Breinig S., Oswald E., Serino M. (2020). Evolution of gut microbiome and metabolome in suspected necrotizing enterocolitis: A case-control study. J. Clin. Med..

[172] Liu X.-C., Du T.-T., Gao X., Zhao W.-J., Wang Z.-L., He Y., Bao L., Li L.-Q. (2022). Gut microbiota and short-chain fatty acids may be new biomarkers for predicting neonatal necrotizing enterocolitis: A pilot study. Front. Microbiol..

[173] Ji J., Ling X.B., Zhao Y., Hu Z., Zheng X., Xu Z., Wen Q., Kastenberg Z.J., Li P., Abdullah F. (2014). A Data-Driven Algorithm Integrating Clinical and Laboratory Features for the Diagnosis and Prognosis of Necrotizing Enterocolitis. PLoS ONE.

[174] Vance D., Frese S.A., Casaburi G. (2019). Artificial intelligence accurately predicts necrotizing enterocolitis from the healthy preterm infant gut microbiome. Pediatrics.

[175] Lure A.C., Du X., Black E.W., Irons R., Lemas D.J., Taylor J.A., Lavilla O., de la Cruz D., Neu J. (2021). Using machine learning analysis to assist in differentiating between necrotizing enterocolitis and spontaneous intestinal perforation: A novel predictive analytic tool. J. Pediatr. Surg..

[176] Lin Y.C., Salleb-Aouissi A., Hooven T.A. (2022). Interpretable prediction of necrotizing enterocolitis from machine learning analysis of premature infant stool microbiota. BMC Bioinform..

[177] Gephart S.M., Underwood M.A., Rosito S., Kim J.H., Caplan M.S. (2020). Grading the evidence to identify strategies to modify risk for necrotizing enterocolitis. Pediatr. Res..

[178] Xiong T., Maheshwari A., Neu J., Ei-Saie A., Pammi M. (2020). An Overview of Systematic Reviews of Randomized-Controlled Trials for Preventing Necrotizing Enterocolitis in Preterm Infants. Neonatology.

[179] Chandran S., Anand A.J., Rajadurai V.S., Seyed E.S., Khoo P.C., Chua M.C. (2021). Evidence-Based Practices Reduce Necrotizing Enterocolitis and Improve Nutrition Outcomes in Very Low-Birth-Weight Infants. J. Parenter. Enter. Nutr..

[180] Neu J. (2020). Necrotizing Enterocolitis: The Future. Neonatology.

[181] Gill E.M., Jung K., Qvist N., Ellebæk M.B. (2022). Antibiotics in the medical and surgical treatment of necrotizing enterocolitis. A systematic review. BMC Pediatr..

[182] Bering S.B. (2018). Human Milk Oligosaccharides to Prevent Gut Dysfunction and Necrotizing Enterocolitis in Preterm Neonates. Nutrients.

[183] Orczyk-Pawiłowicz M., Lis-Kuberka J. (2020). The Impact of Dietary Fucosylated Oligosaccharides and Glycoproteins of Human Milk on Infant Well-Being. Nutrients.

[184] Vongbhavit K., Underwood M.A. (2016). Prevention of Necrotizing Enterocolitis Through Manipulation of the Intestinal Microbiota of the Premature Infant. Clin. Ther..

[185] Sánchez C., Fente C., Regal P., Lamas A., Lorenzo M.P. (2021). Human Milk Oligosaccharides (HMOs) and Infant Microbiota: A Scoping Review. Foods.

